# Direct SERS Detection of Nucleic Acids in the Presence of Spermine: A Unified Nanoparticle Platform Allows for the Elucidation of Surface Adsorption Hierarchies

**DOI:** 10.1021/acs.jpcc.5c01965

**Published:** 2025-05-21

**Authors:** Chiara Deriu, Laura Fabris

**Affiliations:** Department of Applied Science and Technology, Politecnico di Torino, 10129 Turin, Italy

## Abstract

Spermine is a polyamine that is ubiquitous to most analytical protocols for the direct detection of nucleic acids by surface enhanced Raman spectroscopy (SERS), where it is either used as a nanoparticle capping species or a sample aggregating agent. An attentive examination of the literature reveals the existence, in the experimental design of past recent works involving spermine and plasmonic nanoparticles, of important confounding factors relative to the surface chemistry of colloids, limiting the reach of the associated mechanistic hypotheses. Our work introduces a thermodynamics-based framework for comparative SERS studies with no confounding bias related to surface chemistry. Such experimental design is enabled by a unified colloidal nanoparticle platform, constituting the surface chemistry baseline for all of the investigated sample preparation scenarios, with and without spermine. The validation of a “minimal working example” analyte for fundamental studies on ssDNA systems is also presented, which introduces the advantage of experimentally and computationally minimizing the system, for accessible DFT-aided vibrational elucidation. The ensemble of thermodynamic and spectroscopic data gathered in this study allows for a reframing of the mechanistic hypotheses relative to spermine-containing SERS samples by decoupling the system into its pairwise interaction equilibria. It is demonstrated that the spermine-nucleic acid interaction is thermodynamically dominant, and that the equilibria that are established at the nanoscale surface in the presence of spermine are independent of the sample preparation order. Our results and methodological approach are key to the development of selectivity-optimized SERS methods for nucleic acid detection, and have a wide interest reach for all of those nano- and biotechnological fields that exploit surface chemistry interactions.

## Graphical abstract

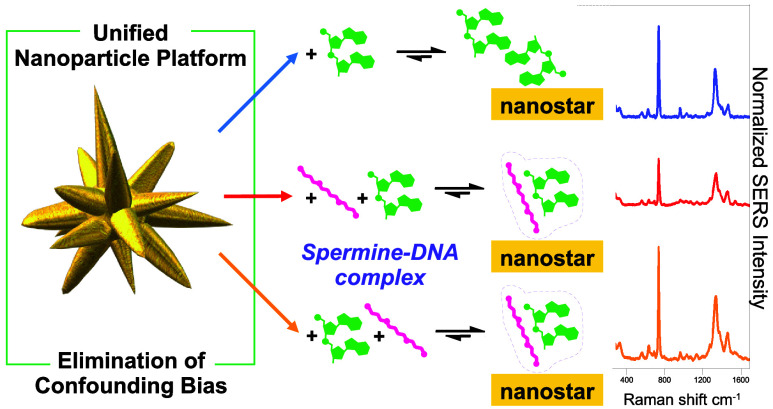

## 1 Introduction

Spermine (*N* ^1^,*N* ^4^-Bis­(3-aminopropyl)­butane-1,4-diamine) is a biogenic^1,2^ polyamine that finds widespread use in a multitude of technological and analytical applications involving the entrapment or detection of nucleic acids, ranging from the vectorization of oligonucleotide-based chemotherapeutic agents or gene therapy^3–8^ to the purification of or from DNA in biotechnological processes,^9–11^ or the detection of nucleic acids by surface enhanced Raman spectroscopy (SERS).^12–26^ In the latter case, spermine is a popular chemical in sample preparation for direct DNA and RNA detection, and it is both utilized as a colloidal nanoparticle capping agent,^24,27^ or as an aggregating agent for traditional negatively charged colloidal nanoparticles.^12,13,19,28^ The most apparent rationale for its use as a capping agent is its polycation nature,^27,29^ for which it is expected to electrostatically attract^19,28^ negatively charged analytes like nucleic acids, favoring their adsorption on the plasmonic surface and confinement within nanoparticle junctions.^20,30^ When utilized as an aggregating agent for anionic nanoparticles, spermine’s polycationic nature is exploited as a charge neutralizer,^19,28^ leading to the formation of aggregate-generated hotspots.^12,28,31^

Following these considerations, spermine could be in principle utilized just like any other adsorbing (poly)­cation for colloidal SERS substrate modification, and thus, for the electrostatic attraction of *any* (poly)­anionic analyte, or for the aggregation process of *any* anionic colloidal system. While this use is certainly possible, as testified by a small number of reports,^32,33^ spermine is in practice very selectively utilized for SERS systems that are *tailored* to the detection of nucleic acids.^12–26^ A biologically rooted rationale for the use of spermine in such applications is that this polyamine is ubiquitously found in eukaryotic cells and is actively responsible for *in vivo* DNA condensation and many other structure- and stability-related phenomena involving nucleic acids.^2,34^

For this reason, many biochemistry-centered experimental^35–41^ and computational^42–49^ studies have been performed since the 1960s to elucidate the chemical basis of the spermine/nucleic acid interaction, with particular attention to genomic DNA and its double stranded (ds) conformations. A very wide range of X-ray methods,^35,36^ NMR,^48^ molecular dynamics,^42–49^ thermal denaturation and calorimetry,^37,50–52^ UV spectroscopy,^38,53^ and even Raman spectroscopy studies^39–41^ all converge toward the identification of an electrostatic attraction between the negatively charged phosphate backbone of DNA and the positively charged amino groups of spermine as the main cause of this interaction. While electrostatic attraction is often emphasized as the primary driving force behind the formation of the complex,^39,54,55^ over the years, improvements in the experimental and simulation methodologies have allowed to highlight at an increasingly atomistic level the coparticipation of nonelectrostatic forces, and thus, a degree of sequence specificity in the interaction between the two species.^38,44,48,49,51,53,56^ Spermine is observed to reside in the grooves of dsDNA, with residence times and binding strengths that are a function of structural parameters, and thus, nucleobase sequence and water solvation conditions.^38,44,47,49,53,56^ Similar sequence- and structure-specific interactions are also extensively documented for RNA.^34^

In the SERS literature, probably as a result of the historical prominence^39,49,54,55^ of the electrostatic model in the biochemistry literature, only electrostatic interactions are systematically reported to explain the physicochemical basis of spermine-DNA complexes:^12,19,25,27,28^ The polyamine is frequently reported to neutralize the phosphate backbone of nucleic acids,^13,19,28^ acting as an “electrostatic glue”.^18,25,31^ A single exception to such an explanation trend is found, in which an additional role of spermine as a “normalizer of affinity levels”^19^ is hypothesized upon observing that the differences in the SERS profiles of 21-mer homopolymeric single-stranded (ss) DNA sequences obtained with anionic colloidal nanoparticles and magnesium sulfate aggregation are leveled out, both in terms of frequencies and intensity, when aggregation by spermine is performed instead.^19^ More pictorially, common schematic depictions see the polyamine either forming positively charged nanoparticle junctions hosting nucleic acids,^14,16,20^ or bridging units interposed between the polyanionic analyte and the negative surface charges of nonaggregated nanoparticles.^19,28,30^

The latter schematic was utilized by Torres-Nuñez et al.,^19^ who proposed that a layered configuration of the type “metal/adsorbed anions/spermine/nucleic acid” is adopted by SERS samples systems based on spermine-capped silver nanoparticles (Ag/Sp^27^), as well as in those instances in which the nucleic acid and spermine are premixed, and then added to an anionic nanoparticle system. However, the latter case is not reported experimentally by the authors, and the presented instances of sample preparation in which aggregation by spermine is performed as the last step, after prior adsorption of free ssDNA targets, are instead not mechanistically discussed.^19^ It was furthermore proposed that, depending on the specific affinity of the nucleobase(s) for the metal surface, a competing, spermine-independent mechanism coexists, where a displacement of preadsorbed anions and a direct nucleic acid adsorption via nucleobase-metal interaction occur.^19^

To this end, it should be stressed that the presence of plasmonic nanoparticles cannot be realistically treated as simple (negatively) charged entities within the SERS sample system,^57^ for two different reasons. The surface of colloidal nanoparticles is a dynamic landscape that is populated by adsorbed species^58,59^ that can either *coadsorb* or be *displaced* by an incoming species (i.e., analyte and/or aggregating agent), depending on the thermodynamic equilibria characterizing the system.^60^ Second, but not less importantly, gold and silver nanoparticles are reported to have non-negligible dispersive and coordinative interactions with both aromatic (e.g., nucleobases) and nitrogen-containing (e.g., polyamines, nucleobases) species, respectively.^61–63^ As a result, the interaction of spermine and nucleic acids *in the presence* of a third *interacting* entity, that is, the colloidal nanoparticle itself (i.e., the plasmonic metal and its adsorbates), is realistically more complex than a direct translation of what happens between nucleic acids and spermine as a dyadic system.

Furthermore, it has been historically challenging to access true nanoparticle controls for fine-level studies of the influence of each individual component in a SERS sample system (i.e., metal, preadsorbed species, analyte, aggregating agents). For example, Torres-Nuñez et al.^19^ observe that spermine-capped and spermine-aggregated samples yield different spectral profiles, suggesting that adsorbed, and thus surface-restrained, spermine molecules have a different behavior toward nucleic acids compared to free spermine molecules in solution. While this explanation could be true, it should be pointed out that none of the compared systems in the study can be utilized as a *true* control or baseline sample, because all of the utilized colloidal nanoparticles were obtained using different syntheses and preparations (i.e., hydroxylamine-reduced^64^ vs citrate-reduced^65^ and halide-activated^66^ vs sodium borohydride-reduced and spermine-capped^27^ silver nanoparticles), which realistically impart differences in their surface chemistry, even after spermine aggregation. Because surface chemistry has a key role in modulating the SERS effect and the resulting spectrum,^57^ these differences can constitute confounding factors, and as such, they cannot be definitively teased apart to draw mechanistic conclusions.

In this paper, a single nanoparticle platform is leveraged to establish a fair comparison paradigm for the direct SERS detection of nucleic acids in the presence of spermine, either as a capping or as an aggregating agent, and extract physicochemical information that is instrumental to the rational development of SERS methods with optimized sensitivity and specificity. The as-synthesized surface of the chosen nanostars has been extensively characterized,^57^ and its use for SERS-related fundamental adsorption studies has been previously validated.^57,60^ The nanoparticle platform is a natively negatively charged, surfactant-free, gold–silver nanostar^57,67,68^ that is sparingly and weakly capped,^57,69^ such that it can be functionalized by common adsorbing ligands, including spermine,^24^ by simple postsynthesis addition,^57^ thus providing us with a spermine-free and spermine-bearing system using *the same* nanoparticle synthesis. By maintaining the nanoparticle synthesis constant, the confounding bias related to the baseline nanoparticle surface chemistry is eliminated. Hence, the resulting experimental design poses fair grounds to “isolate”, elucidate, and compare the different pairwise interactions that are established, as a function of sample preparation order, in the SERS sample, where the thermodynamic equilibria dictate the adsorbate landscape, and thus, the SERS spectrum that we obtain.

The thermodynamic framework with which we conceptualize SERS samples is thus exploited to address fundamental questions that are still open, such as: What happens if spermine is introduced after prior nucleic acid adsorption ―does the surface adsorbate change with respect to the capping agent-induced one? Does the presence of spermine alter the native adsorption behavior of nucleic acids on plasmonic nanoparticles, or does it only result in an adsorption facilitation by charge neutralization, without altering the nucleic acid’s inherent interaction with the metal? Because of the tight link between SERS spectral quality (i.e., frequency and relative intensity) and surface chemistry, answering these questions is instrumental to tailoring sample preparation not only for optimized sensitivity, but also specificity, whereby the ultimate performance push for SERS-based nucleic acid (i.e., *genetic*) testing would be ensured by the maximization of spectral differences among targets bearing the smallest degree of mutation (e.g., single point mutation or epigenetic methylation).

A translational knowledge of such a kind would not be possible without the prior minimization of the degrees of freedom related to specificity itself, and thus, to the analyte. For this reason, we here propose the use of a ssDNA dinucleotide, 5′-AA-3′, as a model analyte for our fundamental studies. We borrowed the concept of “minimal working example” from the coding community,^70,71^ and reduced the ssDNA entity to its minimum functional representation, ensuring that all its characteristic structural elements are retained: the nucleobase, the deoxyribose, a direction with a 5′ and a 3′ end, and, most importantly for spermine-related studies, a phosphodiester bond. The 5′-AA-3′ dinucleotide is here validated as a suitable model analyte for spermine-containing SERS system in *lieu* of longer sequences, thus introducing the advantage of having (i) negligible secondary structure and intermolecular interactions that complicate vibrational elucidation, (ii) a sufficiently small size for density functional (DFT) calculations in aid of vibrational analysis and interpretation, and, again, (iii) retained structural elements that are expected to be crucial for its interaction with spermine and a fair comparison with longer DNA sequences.

The results here presented and the methodological framework are not only expected to enhance our understanding of nanoscale adsorption phenomena that are important to the improvement and real-life actualization of SERS-based genetic testing, but also promise to provide generalizable insight into molecule-nanoparticle mechanisms that can be of interest to other (bio)­nanotechnology-related fields.

## 2 Materials and Methods

### 2.1 Materials

All solutions were made using HPLC grade water. Volumetric flasks were cleaned with aqua regia and rinsed with copious amounts of ultrapure water, followed by HPLC grade water (Supelco). The reagents used for the synthesis of nanostars and SERS samples preparation were tetrachloroauric­(III) acid trihydrate (≥99.9%, Sigma-Aldrich, CAS 16961-25-4), silver nitrate (99.9999%, Sigma-Aldrich, CAS 7761-88-8), l-ascorbic acid (≥99%, Sigma-Aldrich, CAS 50-81-7), and spermine tetrachlorohydrate (Sp; molecular biology grade, Sigma-Aldrich, CAS 306-67-2). Model dinucleotide 5′-AA-3′ was obtained by Metabion as an HPLC-purified, lyophilized sample, which was resuspended using nuclease-free water (Invitrogen), aliquoted, and stored at −20 °C. The concentration was determined spectrophotometrically, utilizing 27,400 L mol^–1^ cm^–1^ as the extinction coefficient at 260 nm, which was determined using the nearest neighbor method.^72–74^ The use of phosphate-containing buffers was avoided to eliminate the risk of parallel phosphate-amino supramolecular interactions that could bias experimental results.^75,76^

### 2.2 Synthesis of Nanostars

A previously reported^68^ and in-depth characterized^57^ synthesis of seedless, surfactant-free gold nanostars (NS) was selected for this study. Briefly, 36 μL of 10^–2^ M HAuCl_4_·3H_2_O and 2 μL of 10^–2^ M AgNO_3_ were added to 1 mL of HPLC grade water and vortexed for 10 s. Subsequently, 6 μL of 10^–1^ M l-ascorbic acid were added all at once to the reactant mixture and vortexed for 20 s. The reaction was performed in as-received glass vials wrapped in aluminum foil. The as synthesized nanostars have a concentration^77^ of 0.055 nM and a maximum extinction at around 700 nm. Nanostar concentration for SERS measurements was obtained via centrifugation at 2320*g*, and resuspension in HPLC grade water to reach a concentration of 0.077 nM.

### 2.3 Nanoparticle Characterization

Morphological characterization of the nanostars was performed using a Thermo Scientific Talos F200X nonaberration corrected Transmission Electron Microscope (TEM), operated at an electron accelerator voltage of 200 kV and fitted with a 16-megapixel complementary metal oxide semiconductor (CMOS) camera (Ceta, Thermo Scientific). Images were acquired in bright field mode using a 30x objective, while elemental mapping by energy-dispersive X-ray spectroscopy (EDX) was performed in Scanning Transmission Electron Microscope (STEM) configuration, and acquired using a 4-quadrant silicon drift detector (SDD). TEM samples were prepared by depositing the colloidal nanostars as 7 μL-aliquots onto continuous ultrathin carbon film coated lacey carbon supported copper grids (Sigma-Aldrich) and observed on a single tilt holder, after prior removal of excess sample by capillarity and drying at ambient temperature and pressure. Optical characterization was performed utilizing a Cary 3500 double beam multicell UV/Visible spectrophotometer fitted with a xenon flash lamp operating at 250 Hz, a double out-of-plane Littrow monochromator, and a silicon photodiode detector. Samples were transferred to 700 μL 1 cm optical path quartz cuvettes (Hellma) and analyzed across the 200 to 900 nm range with a resolution of 1 nm.

### 2.4 Raman Spectroscopy

All Raman spectroscopy measurements were performed using a WITec alpha300 Apyron confocal microscope, fitted with (i) a lens-based, 600 mm focal length ultrahigh throughput spectrometer (UHTS) and a Peltier-cooled back-illuminated electron multiplying CCD (EMCCD) camera for measurements in the visible range, (ii) and a lens-based, 400 mm focal length UHTS and a Peltier-cooled back-illuminated deep depletion CCD camera for measurements in the near-infrared range. The reference Raman spectra were obtained after prior deposition of 1 to 2 μL of the dinucleotide stock solution in nuclease-free water at a concentration in the order of 0.1 mM onto an aluminum foil-wrapped glass microscope slide. The aluminum foil eliminates the glass background from the microscope slide and ensures no spurious contributions are introduced in the resulting Raman spectrum of the analyte. Due to the sporadic presence of trace lipidic residues from the foil manufacturing process (Figure S1), the aluminum foil was found to necessitate a cleaning treatment prior to its use as a Raman-inactive substrate. Such cleaning was achieved with a mixed Ar/O_2_ (70:30, 60 W) plasma treatment at 0.5 mbar pressure for 3 min. This treatment eliminates the residues associated with the lipidic contaminant, producing a surface that has increased wettability, increased apparent roughness, and overall lack of interfering Raman signatures. After sample deposition and drying, the dinucleotide residues were assumed to have crystallized with no waters of hydration and were examined under 532 and 785 nm illumination with a 100× objective and a 300 g/mm grating. Acquisition parameters were individually optimized for each excitation wavelength: 30 s integration time, 2 accumulations, and a power of 5 mW at the laser fiber corresponding to 3.84 mW at the objective for measurements at λ_exc_ 532 nm; 30 s integration time, 5 accumulations, and a power of 70 mW at the laser fiber corresponding to 47.47 mW at the objective for measurements at λ_exc_ 785 nm. A similar procedure was performed for the Raman study of the dinucleotide-spermine samples. In more detail, mixtures of spermine tetrahydrochloride and the dinucleotide (molar excess of 2:1 to avoid precipitation), were incubated overnight, and then deposited and let dry on microscope slides, as described above. Because it was observed that no excitation effect exists for the dinucleotide (Figure S2), an excitation of 532 nm was selected for these measurements; the choice was made because the combination of a 532 nm excitation and a 300 g/mm grating allows to investigate the widest spectral range in the least amount of overall exposure time (i.e., a single acquisition for the ∼200–3400 cm^–1^ range with no spectral stitching) and with a maximized signal-to-noise ratio (S/N). The acquisition parameters were individually optimized for this series of measurements and were: 15 s integration time, 3 accumulations, and a power of 2 mW at the laser fiber corresponding to 1.55 mW at the objective. Spectral resolutions for all measurements acquired with the 532 nm excitation and 300 g/mm grating is 3.01–2.44 cm^–1^ across the 200–2000 cm^–1^ range, while for the 785 nm excitation and the 300 g/mm grating is 1.88–1.36 cm^–1^ across the same range. Detailed spectral resolutions as a function of individual reference wavenumbers are reported in Table S1.

### 2.5 Surface-Enhanced Raman Spectroscopy (SERS)

SERS measurements were performed utilizing the same Raman confocal microscope utilized for traditional Raman measurements. Because the nanostars utilized for the SERS measurements reported in this paper have a plasmon maximum around 700 nm (Figure S3), the laser line at 785 nm was chosen as the excitation wavelength for these experiments. The optical power at the laser fiber was 88 mW, which corresponds to a laser power before the objective of 59 mW, while the integration time and accumulations were 5 s and 10, respectively. Measurements were performed in solution, with a 10x objective, at a room temperature of 20 °C. Samples were prepared according to three different sample preparation orders: (i) Nanostars + model analyte 5′-AA-3′, (ii) nanostars capped by spermine + model analyte 5′-AA-3′, and (iii) nanostars + 5′-AA-3′ + spermine. Representative background spectra of the nanostars in the presence and absence of spermine are reported in Figure S4. All samples were equilibrated for 2 min and transferred into a squared section glass capillary (VitroCom). For *all* SERS measurements, the final NS concentration was 0.07 nM, and a ratio of 6:1 NS:analyte was used; when it was present in the preparation, spermine was maintained at a final concentration of (6.7)­10^–5^ M for all samples. This concentration was chosen on the basis of electrophoretic light scattering (ELS) titration data reported in this paper, as it was sufficient to achieve a positive charge on the nanostar, without saturating its surface or producing unstable samples during measurements. A more detailed explanation of this choice can be found in the Supporting Information (Figure S5). In analogy to what has been reported for traditional Raman measurements acquired with the 785 nm laser line and the 300 g/mm grating, the spectral resolution is 1.88–1.36 cm^–1^ across the 200–2000 cm^–1^ range (Table S1). All SERS measurements for the adsorption isotherm studies were performed in randomized order to avoid time effect bias, and all the resulting spectra were baseline-corrected (WITec Project SIX Plus, shape function) and normalized by the intensity of the metal–Cl stretching band at ∼230 cm^–1^. Nonlinear fitting of isotherm data was performed using OriginPro 2024 and the built-in Levenberg–Marquardt algorithm.^78–80^

### 2.6 Density Functional Theory (DFT) Calculations

DFT calculations consisted of the optimization of the geometry and calculation of the Raman frequencies of 5′-AA-3′, which was modeled as its fully protonated form, as the calculated spectra was primarily utilized to assist in the vibrational analysis of the Raman spectrum of the molecule in the solid phase, in the absence of salts or buffering agents. An initial geometry guess was obtained using Q-Chem 5.4,^81^ with the B3LYP^82,83^ hybrid exchange correlation functional and 6-311G** ^84–86^ basis set.^60^ All subsequent DFT calculations were performed using Gaussian 16 ^87^ at the same level of theory. MultiWFN^88^ was used to convert Raman activities (ν_exc_ = 12738.85 cm^–1^; T = 298.15 K) and to model the calculated frequencies into a simulated spectrum by using a standard broadening Lorentzian function with a width at half-maximum of 20 cm^–1^. Molden^89,90^ was used to visualize the optimized geometry and assign the vibrational modes.

### 2.7 Electrophoretic Light Scattering (ELS) Titrations

Electrophoretic mobility and conductivity measurements of the colloidal formulations were performed via ELS using an Anton Paar Litesizer 500 fitted with a 658 nm single frequency 40 mW laser diode and operating at a 15° detection angle, and a folded capillary Omega 225288 cuvette (Anton Paar). Standard operating procedures for both measurements and data treatment were set up following a previously published method.^57^ Briefly, water was set as the dispersant and the sample viscosity was assumed to be the same as the dispersant viscosity. Three NS batches per spermine tetrahydrochloride concentration point were analyzed as 600 μL aliquots, each in triplicate (N = 9), where each measurement consisted of 100 subruns. The concentration of spermine tetrahydrochloride in the final formulation ranged from a minimum of (4.8)­10^–7^ M to a maximum of (9.5)­10^–4^ M. The pH ranged from 4.0 to 4.5 for all analyzed spermine tetrahydrochloride concentration points, thus ensuring a constant nominal 4+ charge on spermine, as the lowest p*K*_a_ of spermine is reported to be 8.1.^29^ Sample equilibration times were chosen based on the difference between room temperature and the selected temperature for the measurements (*T* = 298.15 *K* = 20 °C) as 1 min/1 °C difference; the average equilibration time was 3 min. Temperature control was ensured by the built-in thermostated cuvette holder of the instrument. The dispersant’s viscosity, refractive index, and dielectric constant are temperature dependent and are reported in Table S2. The viscosity and dielectric constant were obtained by published tabulated data,^91,92^ while the refractive index was calculated utilizing the method published by Bashkatov and Genina.^93^ The ζ potential values were derived from the measured electrophoretic mobilities, according to the equation:

1 $$\mu =\varepsilon_{r}\varepsilon_{0}\eta \zeta \;f\left(\kappa a\right)$$

where ε_r_ and ε_0_ are the dielectric permittivity of the dispersant and vacuum, respectively, η is the viscosity of the dispersant, and *f*(κa) is Henry’s function, which has been approximated as follows:

2 $$f\left(\kappa a\right)\approx \frac{2}{3}\left\{1+\frac{1}{2{\left\{1+\left[\frac{2.5}{\kappa a}\right]\left[1+2e\left(\kappa a\right)\right]\right\}}^{3}}\right\}$$

where *a* is the radius of curvature of the nanostructure expressed in meters, and κ is the Debye–Hueckel parameter expressed in reciprocal meters (eqs S1 and S2). This method, originally published by Ohshima,^94^ is valid for all particle geometries and has been already successfully utilized for quantitative studies on colloidal plasmonic nanostars.^57,95^ Similarly to what was reported for citrate,^57^ after prior outlier screening,^96^ all ζ potential values were corrected by a factor corresponding to the lowest spermine titration point that was able to reverse the net charge of the nanoparticle system, and plotted as the average of the resulting absolute difference, Δ|ζ|. In other words, these differences represent the successive increases in surface charge upon addition of spermine, after an initial reverse of the native surface charge. This procedure is to be intended as a normalization, as it ensures that potential confounding effects due to the possible displacement of preadsorbed chloride during the initial phase of the titration are avoided.^57^ Nonlinear fitting of isotherm data was performed using OriginPro 2024 and the built-in Levenberg–Marquardt algorithm.^78–80^

## 3 Results and Discussion

### 3.1 Vibrational Characterization of Model Analyte

The DFT calculation of the Raman frequencies of model analyte 5′-AA-3′ were obtained at the B3LYP/6-311G** level of theory, prior to energy minimization and stability check at the same level of theory. The absence of negative predicted vibrational frequencies indicates that the geometry corresponds to a true energy minimum. The general structure of 5′-AA-3′and the simplified moiety nomenclature are given in the left panel of Figure 1, while the energy-minimized structure is shown in the right panel of the same figure. The energy-minimized geometry of 5′-AA-3′ is an asymmetric top, with rotational symmetry number of 1; the nucleobases lie in almost perpendicular planes and the 5′ phosphate group is predicted to be at hydrogen bonding distance from the ether moiety of the deoxyribose on the 3′ end of the molecule. The optimized Cartesian coordinates are reported in Table S3.

**Figure 1:**
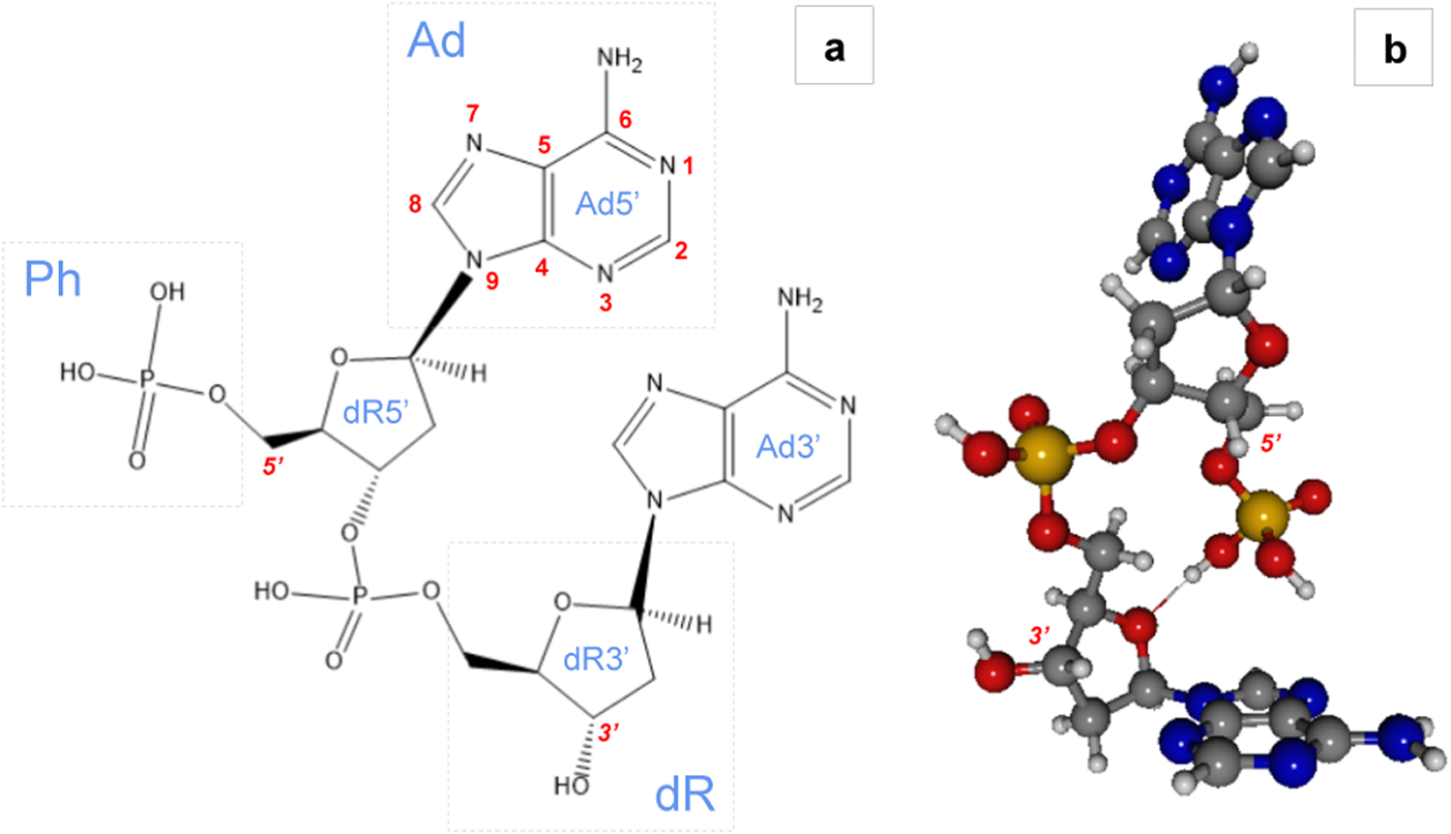
DNA dinucleotide 5′-AA-3′. (a) Structure with standard numbering and nomenclature: adenine (Ad), phosphate (Ph), and deoxyribose (dR). (b) Ball-and-stick 3D model of the DFT-optimized geometry of AA at the uB3LYP/6–311G** level of theory. H atoms are depicted in white, C in gray, O in red, N in blue, and P in yellow.

The level of agreement between the DFT-calculated and experimental Raman frequencies were assessed by the percent error for each unscaled calculated-experimental frequency pair (Table 1). On average, the percent error of prediction is below 3%, denoting an excellent agreement with the experimental data. Notable exceptions are the complex deformation of the phosphate-sugar backbone at 413 cm^–1^ and the skeletal vibration mode at 273 cm^–1^, which are predicted with an error of 6.30 and 8.42%, respectively. These larger, but still acceptable, error values could be attributed to the optimized hydrogen bonded conformation which might differ from the one in which the molecules are actually organized in an ensemble in the solid state; it must in fact be reminded that the DFT calculations here presented refer to the isolated molecule in the gas phase.^97^

**Table 1:** DFT, Raman (λ_exc_ 785 nm), and SERS (λ_exc_ 785 nm, No Spermine) Bands of 5′-AA-3′, with Vibrational Assignments and DFT-Raman Percent Error^a^

DFT (unscaled)	mode #	assignment	Raman	% error prediction	SERS (NS/AA)
1663	174	δ_s_(NH_2_)Ad5′; ν_as_(N10-C6-C5)Ad5′	1634	1.77	
1620	173, 172	ν(N3–C4)Ad; ν(C5–C6)Ad3′; ν(N7–C8)Ad3′; ν(N1–C6)Ad5′	1575	2.86	1555
1526	169, 168	δ(C8–H)Ad; ν(N7–C8)Ad; δ_s_(NH_2_)Ad	1505	1.40	1500
1504	165, 164	δ(C2–H)Ad; ν(C2–N3)Ad3′; ν(N1–C6)Ad	1477	1.83	1459
1487	163, 162	δ_s_(H–C2–H)dR	1457	2.13	
1458	161	γ(C1–H)dR5′; ν(C4–N9)Ad5′; ν(N7–C8)Ad5′	1424	2.39	1397
1445	159	γ(C1–H)dR3′; ν(C4–N9)Ad3′; ν(N7–C8)Ad3′	1412	2.34	
1427	156	γ_w_(H–C5–H)dR5′; γ(C4–H)dR5′	1370	4.16	1369
1364	149, 148	ν(C2–N1)Ad; ν(C5–N7)Ad; γ(CH)dR	1333	2.33	1323
1327	141, 140	δ(CH)Ad5′; γ(CH)dR5′; γ_w_(CH)dR3′; δ(CH)dR	1304	1.76	
1270	131, 130, 129	γ_t_(CH_2_)dR; γ(CH)dR; δ_r_(CH)Ad; δ_r_(NH_2_)Ad	1250	1.60	1242
1238	128, 127	γ_w_(CH_2_)dR; δ_r_(NH_2_); ν_s_(N1–C2–N3)Ad; ν(C5–N7)Ad; ν(C8–N9)Ad		0.96	
1218	125	γ(C8–H)Ad3′; δ(CH)dR3′	1215	0.25	
1191	124	γ_w_(H–C2–H)dR5′; δ_r_(H–C5–H)dR5′; ν(C4–N9–C8)Ad5′; ν(C2–N3)Ad5′; ν(P=O)Ph	1163		
1150	122	δ(CH)dR3′	1129	1.86	1120
1118	120, 119	ν(C3′–O)dR3′; δ_t_(H–C2’–H)dR; δ_r_(CH_2_)dR; ν_as_(C–O–C)dR3′; ν(P=O)Ph	1094	2.19	
1092	116, 117	ν_as_(C–O–C)dR3′; δ_r_(CH_2_)dR5′; ν(C–O)phosphodiester; δ(OH)Ph5′; δ_r_(NH_2_)Ad5′; δ(C8–H)Ad5′; ν(C4–N9)Ad5′	1065	2.54	1068
1048	109	δ_r_(NH_2_)Ad3′; γ_w_(H–C2–H)dR3′	1009	3.87	1026
1039	108	δ_r_(H–C2–H)dR3′	994, sh	4.53	
1004	103	δ_r_(NH_2_)Ad5′; δ_r_(CH)dR5′; δ_r_(H–C5–H)dR5′	948	5.91	957
944	96	ν_as_(O–P–O)Ph5′; δ(ring)dR	902	4.66	
915	94	ν_a_(O–P–O)Ph; ν(O–P–O)phosphodiester; δ(ring)dR	872	4.93	
844	86	δ_r_(CH_2_)dR	830	1.69	
809	83	ν_s_(O–P–O)Ph; δ_r_(CH_2_)dR; ν(O–P–O)phosphodiester	797	1.51	788
780	82	ν(Ph)5′; δ(C–O)phosphodiester; γ_t_(H–C2–H)dR5′; δ_s_(N9–C1–C2)dR5′; δ_r_(CH_2_)dR5′	784	0.51	
727	76, 75; [79, 78]	β(ring)Ad; [δ(ring)dR; δ(C–O–P–O); γ(OH)Ph5′]	729	0.27	730
			685		688
665	72, 71	γ(ring)Ad; γ(O–H)Ph5′	656	1.37	660
636	70	δ(ring–O–P)dR5′–Ph	616	3.25	620
593	68, 67	γ(C2–H)Ad; δ(N1–C6–N)Ad; δ(N3–C4–N9)Ad; δ_r_(CH_2_)dR; δ(P–O–C3′)dR	565	4.96	555
584	66	γ(C2–H)Ad3′; δ(ring)Ad3′		3.36	
533	59, 58, 57	δ(C5′–O–P); δ(C3′–O–P); δ(ring)Ad	531, 505 sh	0.38	
481	54	δ(Ph)	460	4.57	467
459	51	γ(Ph)	438	4.79	
439	49	deformation Ph-dR backbone	413	6.30	422
384	45	γ(dR)	373	2.95	
333	40	skeletal vibrations	325	2.46	323
296	37	skeletal vibrations	273	8.42	
243	32	skeletal vibrations	234	3.85	
		ν(metal–Cl)			229

a Abbreviations: ν = stretching (as = asymmetric; s = symmetric); δ = in plane bending (r = rocking; s = scissoring); γ = out of plane bending (w = wagging, *t* = twisting); β = breathing. Ad = adenine; dR = deoxyribose; Ph = phosphate (see Figure 1); sh = shoulder.

In the fingerprint region, the correlation between the relative intensities of bands in the experimental and calculated Raman spectra is acceptable, with well-predicted main vibrational modes at 729 cm^–1^ and in the 1400–1200 cm^–1^ region and few notable exceptions, both at low wavenumbers (i.e., the skeletal vibration mode at 234 cm^–1^) and at higher wavenumbers (i.e., the at 1505 cm^–1^), as observable in Figure 2. Again, discrepancies can be attributed to possible differences between the real (solid phase, ensemble of molecules) and the optimized (gas phase, isolated molecule) geometries of the analyte, which can involve changes in the polarizability of specific bonds, thus resulting in differences between the actual and the predicted Raman intensities.

**Figure 2:**
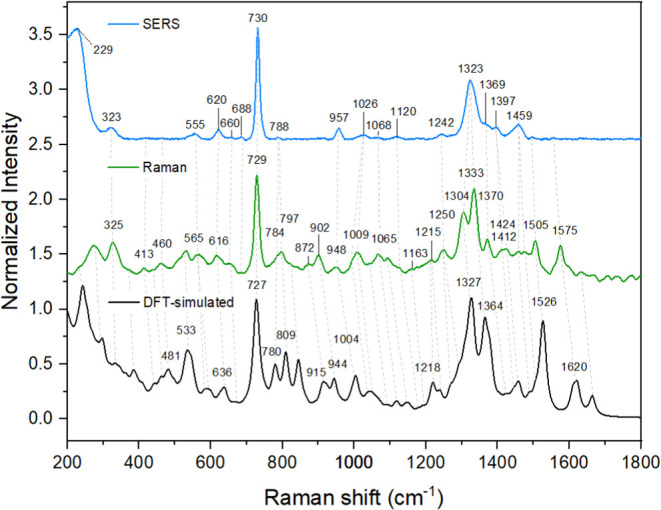
SERS (*top*, *blue*), Raman (*center*, *green*), and DFT-simulated Raman (*bottom*, *black*) spectra of 5′-AA-3′. The SERS spectrum was obtained by direct adsorption of 5′-AA-3′ on colloidal nanostars, at a final analyte concentration of 1.63­(10^–4^) M. Both Raman and SERS spectra were obtained using λ_exc_ 785 nm. All intensities have been normalized to the ring breathing mode of adenine at ∼730 cm^–1^ and are expressed in arbitrary units.

The assignment of vibrational modes to the bands observed in the experimental results was performed on the basis of the calculated frequencies (Table 1). The bands with highest intensity in the normal Raman spectrum of 5′-AA-3′ are those assigned to the adenine nucleobases and, to a lesser extent, to the deoxyriboses. The sharp band at 729 cm^–1^ is mainly assigned to the ring breathing mode of adenine, with minimal bending contributions by the phosphate-sugar backbone at higher wavenumbers, while the other major characteristic bands at 1333 and 1304 cm^–1^ and the medium intensity pattern at 1575–1477 cm^–1^ are assigned to a rich collection of CN and CH stretching and bending modes of the nucleobase, as well as to in-plane and out-of-plane bending modes of the deoxyriboses. The stretching modes of the two phosphate groups are observed at 1163, 1094, 902, 872, 797, and 784 cm^–1^, while the in-plane and out-of-plane bending modes are at 460 and 438 cm^–1^, respectively. The former stretching mode, ν­(P=O), at 1163 cm^–1^ appears as a contribution to a rich ensemble of modes relative to both the nucleobase and the sugar, and is downshifted compared to literature data of longer DNA sequences and model organophosphates, which typically report frequencies around 1200 cm^–1^.^98,99^ Such a difference could be caused by either a discrepancy between the calculated structure and the real structure, which could lead to possible assignment inconsistencies for this specific band, or by an actual conformational-related downshift that is peculiar of the experimental molecule and is correctly described by the DFT results. This band is indeed expected to be particularly sensitive to conformational parameters.^99^ Finally, no comparison between 5′-AA-3′ in the solid state and in solution was possible, as the Raman spectrum of the dinucleotide in aqueous solution at the highest available concentration (1.14 mM) is unfortunately too diluted, and barely yielded any useful signal for a thorough vibrational characterization (Figure S6)

Figure 2 also reports the SERS spectrum of 5′-AA-3′, which was obtained by direct adsorption of the dinucleotide on the colloidal nanostars, in the absence of any additional agent (i.e., spermine). In terms of band position, the SERS spectrum shows a profile that is overall analogous to the one obtained by traditional Raman, suggesting the absence of any significant chemisorption event. Notable differences between the SERS and the Raman spectra are observed in the intensity domain, in that the bands arising from major (in-plane) adenine contributions dominate the profile, while bands assigned to the phosphate backbone tend to have relative intensities that are much lower than those recorded in traditional Raman. Such differences in relative intensity could be due to an orientation of the adsorbed 5′-AA-3′ on the surface that favors the enhancement of the nucleobase modes; the adsorption of oligonucleotides to plasmonic metals is indeed frequently reported to be driven by lone pair interactions at the level of the endocyclic (i.e., adenine’s N7) and exocyclic (i.e., adenine’s NH_2_) nitrogen atoms of the nucleobases.^100,101^

### 3.2 Validation of 5′-AA-3′ as the Model Analyte

The vibrational analysis discussed in the previous section shows that the 5′-AA-3′ dinucleotide exhibits a Raman (and SERS) spectral profile that is overall in line with those reported for longer, single-stranded sequences, with frequency differences arising from the base composition, sequence, length, and conformation, as it has long been known.^26,98,102,103^ However, similarities in the spectral profile of the bare molecule do not guarantee a similarity in the interaction behavior with spermine. For this reason, a Raman study of the 5′-AA-3′/spermine mixture was deemed necessary prior to utilizing 5′-AA-3′ as a “minimal working example” and model analyte. Figure 3 shows three representative Raman spectra (λ_exc_ 532 nm) of 5′-AA-3′, spermine tetrahydrochloride, and their mixture. The latter exhibits a spectrum that is characterized by changes in terms of both relative intensity and band position, which are most evident in regions where there is no overlap with the signals of the individual mixture components. An example of such a case is the region from 700 to 770 cm^–1^ reported in the right panel of Figure 3, in which a shoulder at about 755 cm^–1^ is evident where no other band from the individual molecules is, suggesting the formation of a complex. As opposed to a physical mixture, which results in a spectral profile that is the overlap of those of the individual mixture components and can thus be modeled by their linear combination (Figures S7 and S8), a complex results in the formation of new bands and/or in significant alterations of the relative intensities, due to the modification of the electronic structure of the functional groups involved in the interaction.

**Figure 3:**
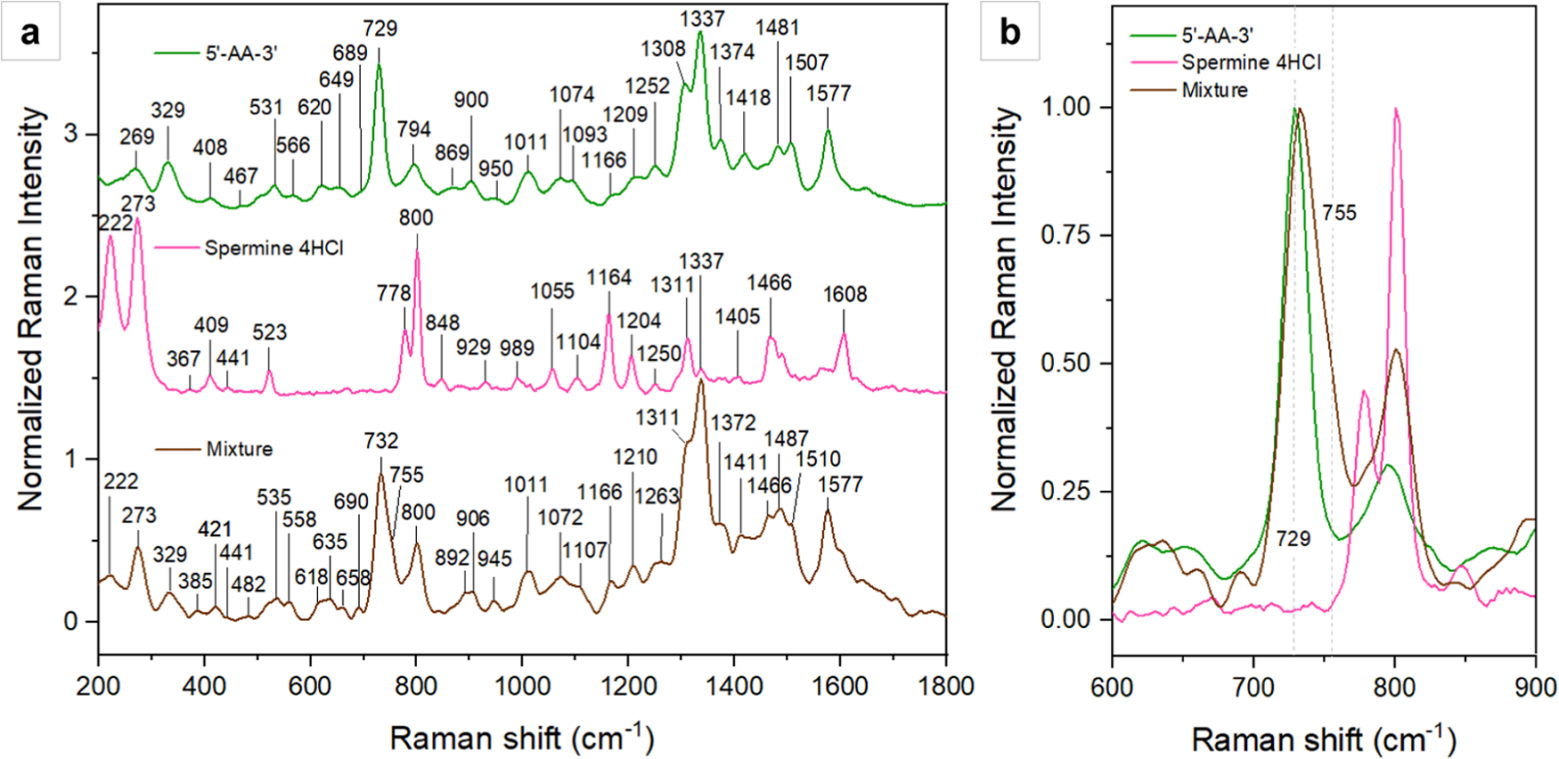
*(a)* Raman spectra (λ_exc_ 532 nm) of the dried residues of 5′-AA-3′ (*top*, *green*), spermine tetrahydrochloride (*center*, *pink*), and their mixture (*bottom*, *brown*). *(b)* An asymmetric broadening of adenine’s ring breathing mode at 729 cm^–1^ can be observed, with shoulder formation around 755 cm^–1^. This shoulder is attributed to an amine-phosphate interaction upon which a 5′-AA-3′/spermine is formed. All intensities have been normalized to the most intense band in the 700–850 cm^–1^ region (∼730 cm^–1^ for both 5′-AA-3′ and the mixture, and 800 cm^–1^ for spermine tetrahydrochloride) and are expressed in arbitrary units.

Significant changes of such a kind can also be observed in the lower wavenumber region of the mixture spectrum: A new band appears at 635 cm^–1^, with an accompanying shift to higher wavenumbers of the neighboring band at 658 cm^–1^; the triplet at 520, 538, and 558 cm^–1^ differs in both intensity and frequency to the triplet at 505, 531, and 566 cm^–1^ observed in the spectrum of the dinucleotide alone, a doublet at 467 and 482 cm^–1^ and a pattern of two weaker bands at 421 and 385 cm^–1^ appear in place of the single bands at 467 and 408 cm^–1^, respectively (Figure S9a). On the higher wavenumber side of the ∼730 cm^–1^ band are also three relevant blueshifts at 1107, 906, and 892 cm^–1^, the latter of which is accompanied by an intensity increase relative to the neighboring band at 906 cm^–1^ (Figure S9b).

At higher wavenumbers in the fingerprint region, the spectrum of the mixture is dominated by the contributions of the nucleobase, similarly to the individual dinucleotide spectrum. Compared to the latter, the changes observed in this region are milder than in the lower wavenumber region, and more likely attributable to band convolution effects rather than definitive electronic structure changes. For example, the pattern in the 1300–1150 cm^–1^ region could be explained as the result of the overlapping presence of the 1252, 1209, and 1169 cm^–1^ bands of 5′-AA-3′ (i.e., complex ensemble of adenine stretching modes and deoxyribose bending modes, Table 1), to the bands at 1250, 1204, and 1164 cm^–1^ of the polyamine (i.e., aliphatic chain twisting modes),^104^ with possible contributions to the distortion of the overlapping pattern by the rocking modes of the NH_3_^+^ groups,^104^ which are likely not characterized anymore by a close hydrohalide environment. The high wavenumber region comprised between 3400 and 2300 cm^–1^ is of more difficult interpretation and assignment. This notwithstanding, it remains clear that the spectrum of the mixture cannot be explained by a linear combination of the two constituting species (Figure S8), thus supporting the hypothesis that 5′-AA-3′ *does* interact with spermine, as reported for longer DNA sequences.^37–41,46^

Interestingly, the majority of the bands that display the most significant changes (<1000 cm^–1^) appear to result from shifts and relative intensity increases of dinucleotide bands that are assigned to the phosphate backbone modes (Table 1), in agreement with previously published Raman data on mixtures of longer DNA sequences with spermine.^39–41^ As a result, the use of the dinucleotide 5′-AA-3′ as a model analyte for our fundamental studies is experimentally supported. As highlighted in the introduction, the abundance of spectral changes in correspondence to phosphate-related Raman bands has been interpreted to be a result of electrostatic interactions (i.e., nonspecific, physical interactions);^39^ however, a number of studies exist where the extent of the electrostatic character of amino-phosphate interactions is re-evaluated, in light of the cues on chemical specificity that emerge from the observation of the strong coupling of these moieties in biologically relevant systems.^75,105–108^

Experiments ranging from docked lysine and arginine residues or phosphatidylethanolamine on phosphatidic acid probed by solid state magic angle spinning ^31^P NMR^106,107^ to polyallylamine-capped silica microparticles with adsorbed inorganic phosphate probed by ζ potential measurements,^75^ or again, polyallylamine-functionalized nanochannels with adenosine triphosphate (ATP) probed by conductometry,^108^ showed that the charge on the phosphate moiety increases upon interaction with positively charged (poly)­amino groups. The chemical basis for this charge increase and consequent stabilization of the amino-phosphate pair has been found in a species-specific, reciprocal p*K*_a_ modulation upon contact, whereby a higher degree of protonation and deprotonation of the amine and phosphate groups, respectively, strengthens the interaction^75,107^ and positively affects the binding affinity.^75^ This behavior, sometimes referred to as the electrostatic/hydrogen bond switch mechanism,^107^ is not observed in control experiments with other divalent polyatomic anions^75^ and is found to characterize various types of phosphates, from inorganic ions to phosphodiesters in ATP,^75,108^ thus strengthening the claim for a generalizable nonpurely electrostatic, chemically specific nature of all (poly)­amino-phosphate interactions.^75,105–108^

These considerations on the existence of a chemically specific character in the amino-phosphate interaction could be utilized to justify the shifts toward higher frequencies that are observed for the phosphate backbone-related modes in the Raman spectrum of the spermine/5′-AA-3′ mixture. Because this dual specific/nonspecific nature of the interaction is similar to that observed for metal cations,^34^ a (loose) parallel can be made with the latter. Strong, nonpurely electrostatic interactions such as those established by Mg^2+^ with ATP, which form an inner shell coordination complex, have been reported to cause an apparent blueshift of the phosphate stretching band, resulting from a concomitant intensity decrease of the nonligated nonbridging phosphate stretching band and the formation of a new [phosphate–ligand] stretching band at higher frequencies.^109^ While a direct structural-spectroscopic translation of such findings cannot be made for our different cation-phosphate system at the level of information that we currently have, it is however reasonable to expect that the discussed chemically specific, nonpurely electrostatic character holding our spermine/5′-AA-3′ complex together will also result in blueshifts of the phosphate modes. The new shoulder at about 755 cm^–1^, on the other hand, could be explained to result from a distortion of the phosphodiester bond upon spermine interaction.

As reported in Table 1, a very minor contribution that is convoluted in the major adenine ring breathing mode at around 730 cm^–1^ is known to include the deformation of the phosphodiester bonds and deoxyribose rings^110^ (i.e., modes number 79 and 78 of our calculations). Therefore, it could be inferred that the interaction with spermine causes an increase in the polarizability, and, thus, in the intensity of this contribution, resulting in the formation of a shoulder. The position of the bands associated with deformations of the phosphodiester-sugar backbone are reportedly very sensitive to conformational parameters, such that they allow for the discrimination of B, A, and Z dsDNA conformations.^111^ Because Z-DNA has a characteristic phosphodiester band centered at 745 ± 3 cm^–1^,^111^ as opposed to B-DNA and A-DNA, which fall at 835 ± 7 and 807 ± 3 cm^–1^, respectively,^111^ and because spermine is known to induce B to Z conformational transitions,^112–116^ it could be hypothesized that the type of structural phosphodiester deformation that is induced by spermine in our dried mixture sample may be similar to the bond conformation in Z-DNA strands.

### 3.3 Thermodynamic Characterization of the Spermine-Nanostar Interaction

In the introduction, we delineated a thermodynamic framework with which all colloidal SERS samples can be schematized and approached for elucidation: The surface landscape of a colloidal SERS sample at equilibrium is the result of the interplay of all of the paired thermodynamic equilibria that characterize its individual components.^60^ Therefore, it is important to quantitatively characterize such equilibria, whenever possible. Our prototypical^12–26^ colloidal SERS sample for the direct detection of nucleic acids can be schematized into three components: the plasmonic nanoparticle, the model analyte 5′-AA-3′, and spermine. While quantitative data on the interaction of (primarily genomic) DNA and spermine exist in the literature,^50–52^ there is at present no counterpart for the spermine-plasmonic nanoparticle pair.

Therefore, the interaction of spermine with the as-synthesized nanostars was quantified via electrophoretic light scattering (ELS) titration of the charged molecule on the nanoscale substrate, following a method originally developed for the thermodynamic characterization of citrate adsorption on the same nanostar system.^57^ ELS or ζ potential titrations of charged adsorbing species on well characterized (*vide infra*) colloidal nanoparticles can indeed be treated as adsorption isotherms, and if they are suitably described by thermodynamically derived adsorption models, they can be utilized to derive thermodynamic quantities such as the affinity constant and the free energy change.^57,117^ The colloidal nanostars selected for this work are surfactant-free and natively sparingly capped, with a native surface charge that is primarily caused by adsorbed chloride ions derived from the gold precursor salt, HAuCl_4_, and a negligible amount of unreacted l-ascorbate anions (apparent *K*_ad_ with gold ∼ 10^2^ M^–1^,^69^ weak interactions), as reported in a previous publication.^57^ This characteristic was demonstrated to make these nanoparticles particularly suitable for use as a “blank substrate” in fundamental studies on the adsorption of postsynthetically added compounds.^57,60^ Their XPS analysis is reported to be consistent with an alloy of gold and silver (the reactant stoichiometry is Au:Ag 18:1),^57^ with no clear pattern of monometallic phase segregation by EDX imaging (Figure S10).

The ELS method extensively reported in Section 2.7 yielded an adsorption isotherm of spermine on the colloidal nanostars, which was best described by a Langmuir-Hill^118,119^ model, with an adjusted R^2^ of 0.999 (Figure 4) and normally distributed residuals (Figure S11). This model is characterized by a cooperativity term, the exponent *n*, which is fixed and independent of the adsorbate saturation conditions:^57,119^

3 $$\Delta {\left|\zeta \right|}_{i}=\Delta {\left|\zeta \right|}_{sat}\frac{{[\mathrm{Sp}]}_{i}^{n}}{K_{d}{\left[\mathrm{Sp}\right]}_{i}^{n}}$$

**Figure 4:**
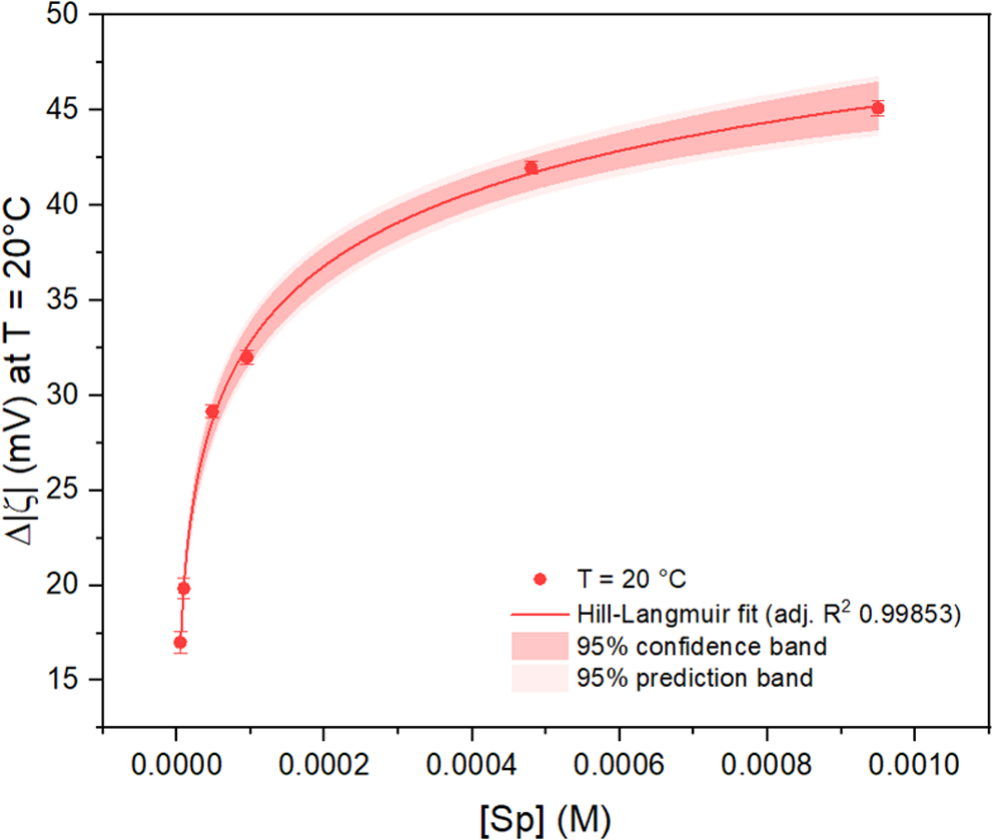
ELS-derived adsorption isotherm (*T* = 20 °C) of spermine tetrahydrochloride (Sp) on colloidal nanostars, fitted to the Hill-Langmuir adsorption model in eq 3. Error bars represent the standard error of the means.

As summarized in Table 2, the polyamine exhibits a surface dissociation constant with the nanostar, *K*_d_, of (1.1 ± 0.5)­10^–4^ M, and a negative cooperativity behavior (exponent *n* < 1; *n* = 0.35 ± 0.03). While a mechanism detailing the negative cooperativity highlighted by the model has yet to be formulated, the phenomenon has been tentatively attributed to solvation effects in water that favor the free molecule rather than the surface complex,^69,120^ or to intermolecular repulsions due to the high nominal charge of the polyamine (i.e., 4+).

**Table 2:** Thermodynamic Parameters for Spermine Adsorption on Nanostars at *T* = 20 °C

*K*_d_	(1.1 ± 0.5)10^–4^ M
*K*_ad_	(9 ± 4)10^3^ M^–1^
Δ*G*_ad_	–5.3 ± 0.3 kcal/mol
cooperativity (*n*)	0.35 ± 0.03

Because the Langmuir-Hill model is thermodynamically derived, the numerical equivalence between *K*_eq_ and *K*_ad_ holds true, such that^57,117^

4 $$\Delta G_{\mathrm{ad}}=-\mathrm{RT}\;\ln\left[K_{\mathrm{ad}}\left(1\;M^{-1}\right)\right]$$

where *K*_ad_ corresponds to 1/*K*_d_. The calculated Δ*G*_ad_ for the spermine-nanostar system is −5.3 ± 0.3 kcal/mol at a temperature of 20 °C, with a *K*_ad_ in the order of 10^–3^ M^–1^, which can be interpreted as an indication of a weak chemisorptive phenomenon between the capping agent and the nanostar surface,^57^ in line with expected binding energy trends^63,121^ that are explicable through hard–soft acid–base (HSAB) theory.^63,122,123^ Computational adsorption studies typically model alkylamines as neutral species,^120,121,124,125^ with adsorption on coinage metal surfaces such as the common Au(111) found in nanoparticles taking place as lone pair-driven coordinative N–Au interactions of varying strength, depending on basicity and steric hindrance.^120^ However, in our case, the pH conditions that were maintained throughout the ELS titration impose four positive charges per spermine molecule (see Section 2.7), and thus, the lone pair of nitrogen is realistically unavailable to interact with the gold and silver surface of the nanostars. For this reason, computational studies on charged amino functionalities such as those of basic amino acid residues on coinage surfaces^126,127^ could provide a more suitable theoretical term of comparison to formulate energetics-based hypotheses on the adsorption mechanism. Stemming from this parallel with basic amino acid literature,^126,127^ it could be hypothesized that the charged amino groups of spermine interact with our gold–silver nanoparticle surface more weakly than neutral amino groups due to the unavailability of the lone pair on nitrogen atoms,^126^ and have a possible adsorption mode that is enabled by the mediation of surface-adsorbed, first solvation shell water molecules.^126,127^

### 3.4 SERS Isotherms as Tools for Adsorption Competition Elucidation

Our methodological approach requires the decoupling of one more binary system from the investigated subject triad of nanoparticle + nucleic acid + spermine —the nucleic acid-nanoparticle pair. This pair was investigated using SERS isotherms because they have the potential to not only provide thermodynamic qualitative and quantitative information,^128–130^ but also molecular information via spectroscopic elucidation. SERS isotherms are obtained by simply plotting the normalized intensity of a reference band of the analyte as a function of its concentration in the colloidal sample —in other words, they are a different way to look at a regular calibration curve.^128,130^ Thanks to the inherent surface origin of SERS spectra,^57,129^ these data can be suitably fitted by adsorption models to characterize and quantify the adsorption behavior of the analyte,^128–130^ in the same way as it was done for the ELS titration data discussed in the previous section.

From a strictly vibrational point of view, the nanostar-analyte system, herein referred to as NS + AA, has already been briefly introduced in Section 3.1, where commonalities and differences between the resulting SERS spectrum and its solid phase reference Raman counterpart were discussed. Briefly, the differences can be summarized as an (expected)^100,101^ amplification of the nucleobase modes relative to the intensities of the phosphate and sugar modes. These characteristics are maintained throughout the investigated concentration range, and no concentration-dependent shifts or intensity changes are observed (Figure S12).

The NS + AA SERS isotherm data points are shown in Figure 5 as blue squares. It can be observed that these data points seem to follow a sigmoidal behavior, evidenced by a connecting Akima spline in the graph. This type of behavior could be classified as an anti-Langmuir, S class^131^ adsorption isotherm, in which the formation of adlayers occurs before the saturation of the first monolayer is completed, due to a tendency of the adsorbate to pack vertically,^131^ and a similarity between the interaction constants characterizing the analyte-metal (A–M) and the analyte–analyte (A–A) equilibria:^132,133^

5 $$A+M\overset{K_{A\;-M}}{⇌}\left[AM\right]$$

6 $$A+A\overset{K_{A-A}}{⇌}\left[\mathrm{AA}\right]$$

7 $$K_{A-M}≅K_{A-A}$$

**Figure 5:**
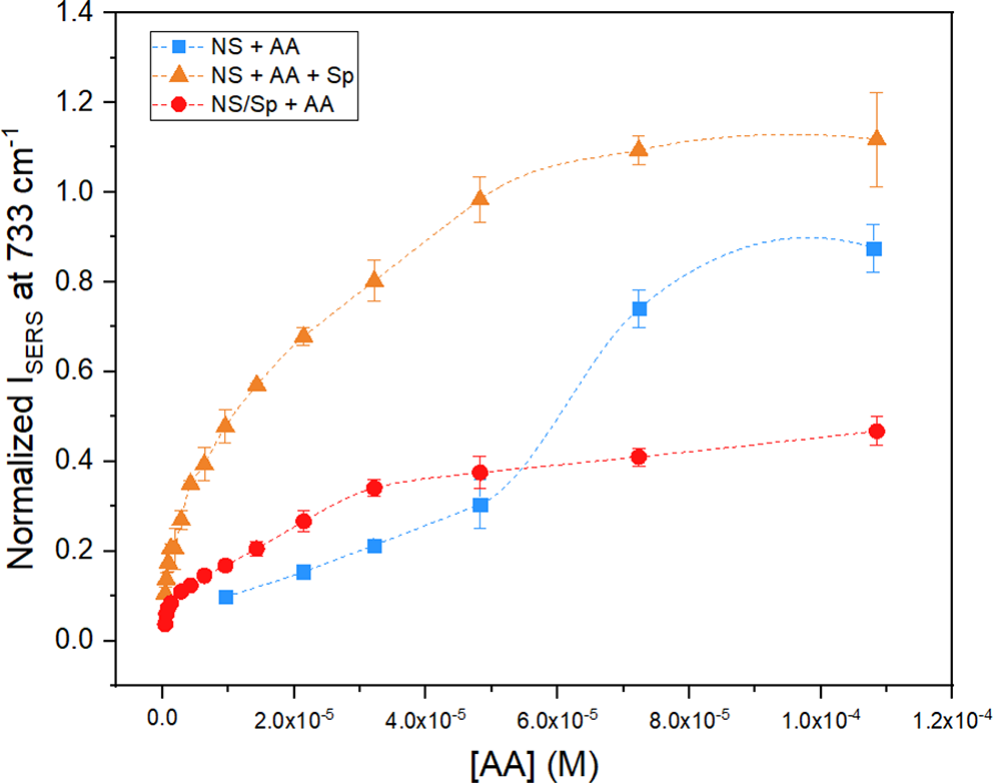
SERS adsorption isotherms of NS + AA (*blue squares*), NS/Sp + AA (*red dots*), and NS + AA + Sp (*orange triangles*). Akima splines are utilized to guide the reader’s eye. While the full behavior at lower concentrations could not be obtained for the NS + AA system, the data suggest that the presence of spermine alters the native adsorption behavior of 5′-AA-3′ on the colloidal nanostars. Error bars represent the standard error of the means.

It is known that nucleobases tend to interact with each other by stacking with non-negligible interaction energies;^134–136^ moreover, it has been experimentally observed that adenine molecules adopt a submonolayer, disordered (i.e., variably tilted), upright conformation on Au(111) surfaces when deposited from saturated aqueous solutions.^135^ Therefore, it is plausible that the adsorption of the 5′-AA-3′ dinucleotide on the utilized plasmonic nanostars in the absence of spermine is mediated by phenomena that are similar to those occurring with the nucleobase alone, resulting in disordered adlayers, ultimately culminating in a sigmoidal SERS isotherm. For the sake of completeness, it should be added that the relatively limited detection range that is attainable with this particular system (the lowest detectable concentration by 0.5 increments was (9.5)­10^–6^ M, Figure S12) does not allow for the verification of the adsorption behavior at truly diluted concentrations. Because of this limitation, there is a possibility that the analyte could behave nonsigmoidally at lower concentrations, and that what is observed in the blue SERS isotherm in Figure 5 is in fact the formation of an adlayer that occurs *after prior saturation* of the first monolayer, as in type L4 isotherms (Figure S13).^131,137^

Regardless of the origin of the observed sigmoidal behavior of the NS + AA system, it can be easily seen that the introduction of spermine in the system *changes* the native adsorption behavior of 5′-AA-3′ on the reference nanoparticle system (Figure 5, round red and triangular orange isotherm data points). The ternary systems containing spermine, either as a capping agent (NS/Sp + AA) or as an aggregating agent (NS + AA + Sp) display a class L isotherm behavior,^131,137^ in which the more adsorbing molecules are introduced into the system, the more difficult it will be for them to find an available site on which to adsorb. As opposed to class S isotherms, vertical assembly of analyte molecules as formalized by the equilibrium in eq 6 is discouraged by K_A–M_ > *K*_A–A_.

The observed change in the adsorption behavior for the two ternary systems NS/Sp + AA and NS + AA + Sp is also reflected in the SERS profiles, which differ from the one obtained in the absence of the polyamine (Figure 6, *left panel*). Net of differences in the overall SERS intensities, which will be discussed later, both spermine-containing systems exhibit the same frequency and relative intensity differences compared to the SERS spectrum obtained on the *same* colloidal nanoparticles. All frequencies appear blueshifted to varying degrees, with only one exception staying unvaried, the band at 1028 cm^–1^, which is assigned to the rocking mode of adenine’s exocyclic amine, as well as to the CH_2_ wagging of the sugar (Table 1). This absence of blueshift could be tentatively attributed to the fact that adenine’s exocyclic amine is reported to be the preferential moiety for the direct interaction with plasmonic metals,^100,101^ thus remaining unvaried in all systems, with and without spermine. In both spermine-containing systems, the largest blueshifts are observed at 1133, ∼1193 and 1269 cm^–1^, corresponding to a Δ|*v̅*| of 16, ∼13, and 27 cm^–1^, respectively. These bands are assigned to a complex ensemble of modes relative to the nucleobase and deoxyribose, plus some contributions of the phosphate group, suggesting an important and nonlocalized deviation from the molecular geometries and orientation on the nanostar surface that characterize 5′-AA-3′ alone. Another region in which deoxyribose- and nucleobase-related deviations are very evident is between 1390 and 1270 cm^–1^, where the symmetry of the mode centered at 1323 cm^–1^ in the NS + AA system gets inverted and shows a more intense contribution at 1334 cm^–1^, with two shoulders on the lower wavenumber side of the band, at around ∼1326 and ∼1306 cm^–1^.

**Figure 6:**
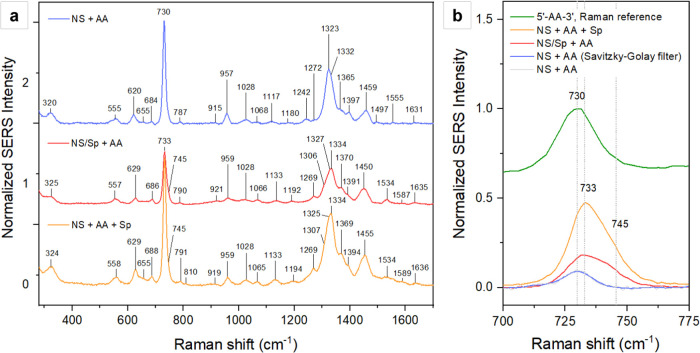
Comparison between the SERS spectra of 5′-AA-3′ obtained in the presence and absence of spermine tetrahydrochloride (Sp), according to different sample preparations. (*a*) The SERS spectrum obtained in the absence of spermine is reported in blue (*top*), the NS/Sp + AA spectrum is reported in red (*center*), and the NS + AA + Sp is reported in orange (*bottom*). The spectral profiles obtained in the presence of spermine are qualitatively equivalent across the fingerprint region, possibly suggesting thermodynamic trends that achieve the same surface adsorbate architecture regardless of the order of addition of sample components. The spectra are on the same scale but stacked for ease of comparison. (*b*) The zoomed-in region between 700 and 775 cm^–1^ shows the formation of a shoulder at 745 cm^–1^ for the spectra obtained in the presence of spermine, similar to what was observed for the Raman spectrum of the spermine-dinucleotide mixture in the solid state (Figure 3). The Raman spectrum of 5′-AA-3′ (*green*) is stacked at the top for reference.

One last notable difference between the SERS spectra of the spermine-bearing systems and the one of 5′-AA’3′ on the colloidal nanostars alone is the formation of a shoulder at 745 cm^–1^, similar to what was described for the Raman spectrum of the spermine/dinucleotide mixture in the solid state. By analogy, this shoulder is interpreted as an increase in polarizability of the phosphodiester deformation mode, likely caused by the association of spermine with the model dinucleotide (Figure 3, *right panel*). Moreover, the spectral resolution of about 1.73 cm^–1^ of the obtained SERS measurements in the region around 700 cm^–1^ (Table S1) is such that a small blueshift of the ring breathing mode of adenine itself can also be called. This shift is in line with the global blueshift trend observed throughout the measured spectral range for both NS/Sp + AA and NS + AA + Sp, suggesting major conformational changes that involve all three constituting moieties of the dinucleotide. Such a moiety-unspecific, generic character of the spectral changes can be in turn interpreted as the likely result of the concomitant action of both spermine *and* the metallic surface.

Even in this case, a within-system comparison of the SERS spectra at the extreme sides of the probed concentration ranges shows equivalence both in terms of frequencies and relative intensities; on the other hand, a comparison of intensity trends between the two systems shows that the overall intensities obtained in the presence of spermine as a capping agent are lower than those obtained when utilizing spermine as an aggregating agent, even though the concentration of all components in the systems are maintained constant. A hypothesis for this behavior could be a synergistic interplay of charge effects and analyte/hotspot ratios. On one hand, the reduction of the interparticle spacing by an overall shift of the colloid charge toward more neutral values possibly occurs more abruptly in a system in which the nanoparticle is more negative (NS + AA), compared to a system in which partial charge reverse has already been “buffered” (NS/Sp); abrupt changes to the surface charge of colloids may induce a less controlled formation of stable aggregates, with possible larger amount of molecules trapped within the resulting interparticle junction hotspots, thus yielding high intensity SERS spectra.

It could be hypothesized that the native mechanism is a multilayer adsorption, possibly sustained by π-stacking, while spermine “regularizes” the adsorption process into an L-type adsorption isotherm, by making the dinucleotide interact with the surface *as a spermine/5′-AA-3′ complex*. The morphology of this complex likely retains a significant level of distortion of the phosphodiester bond that is observed in the absence of nanoparticles in the solid state, as evidenced by the presence of a shoulder at 745 cm^–1^. Due to the S to L adsorption isotherm switch, it is possible to believe that the distorted conformation of the complex discourages the mechanism by which the native multilayer adsorption occurs, for example by steric hindrance and/or a reduction of the conformational flexibility compared to the free dinucleotide. Such a level of “rigidity” can be interpreted in terms of a high degree of stability of the complex, because the surface-interacting species in both NS/Sp + AA and NS + AA + Sp show the same SERS spectrum. In other words, regardless of the pre-established equilibrium, either the metal-spermine or the metal-analyte one, the addition of the third remaining element to complete the triad leads to the formation of a surface adsorbate landscape that has the same identity. For this to occur, the formation of an analyte-spermine complex must be favored at a thermodynamic level.

Our results and the associated interpretation are in contrast with what was hypothesized by Torres-Nuñez et al.,^19^ who reported different SERS spectral profiles upon utilization of spermine as either a capping agent or an aggregating agent, and interpreted these differences as stemming from a diversification of the interaction behavior between preadsorbed, surface-anchored spermine molecules in NP/Sp and free spermine in solution in NP + DNA + Sp samples. Using chemical equilibrium language, the hypotheses formulated by Torres-Nuñez et al.^19^ translate to a hierarchical order of the equilibria that sees the interaction between spermine and the metal as the strongest: *K*_eq(spermine+nanoparticle)_ > *K*_eq(spermine_any_ + DNA)_ and *K*_eq(spermine_free_ + DNA)_ ≠ *K*_eq(spermine_adsorbed_ + DNA)_. However, as noted in the introduction, the nature of the nanoparticle in the two equilibrium scenarios investigated by Torres-Nuñez et al.,^19^ hence its surface chemistry, is different; the “surface-anchored spermine” refers to a system that was obtained by the *in situ* capping of silver nanospheres during sodium borohydride reduction,^27^ while the free spermine is instead added to citrate-reduced and halide-activated silver nanoparticles.^65,66^ In our opinion, these differences cannot be neglected: Different surface chemistries reasonably generate different surface equilibria, and thus, different equilibrium and affinity hierarchies.

The use of the *same* colloidal nanoparticles to compare the SERS adsorption isotherm and spectral data in our experimental design allows for the elimination of the confounding bias caused by the treatment of different surface chemistries as equivalent starting points for the evaluation of adsorption dynamics and the elucidation of the physical meaning of SERS spectra. In this way, a fair comparison is established for the first time, whereby the observed effects can realistically be attributed only to the perturbations that were *intentionally* generated by the experimental design —in this case, the sample preparation order. Opposite to what has been proposed in previous studies,^19^ our results suggest that the affinity of spermine for the model dinucleotide is stronger than all other interactions that take place in a ternary system of colloidal nanoparticle + spermine + nucleic acid, because the adsorption behavior and the SERS spectral profile appears to be independent of the order of addition of the sample components, and thus, they are independent of the order in which the pairwise equilibria are introduced and established.

To quantitatively explore these hypotheses, the lower concentration range of the NS/Sp + AA SERS isotherm data were fitted to an L type isotherm model, the Hill-Langmuir function (eq 3 with *I*_SERS_*i*__ as the independent variable), to extract information about the energetics of the interaction between preadsorbed spermine and incoming 5′-AA-3′ molecules (Figure 7 and Table 3). As it was done for the spermine/nanostar interaction, the desorption constant was directly extrapolated from the fit (adjusted *R* ^2^ of 0.995 and normal residuals), resulting in a *K*_d_ of (6.2 ± 0.7)­10^–7^ M, which corresponds to a *K*_ad_ of (1.6 ± 0.2)­10^6^ M^–1^. This value represents the affinity of the model nucleic acid for a spermine-capped nanoparticle, and is 3.5 orders of magnitude higher than that estimated for the interaction of spermine alone on *the same* nanostars. It is interesting to note that calorimetry-determined affinity constants for the association of genomic and synthetic dsDNA with *free* spermine that can be found in the literature are of similar order of magnitude,^50–52^ with *K*_a_ values for AT-rich and AT-only dsDNA ranging from (6.97 ± 0.04)­10^6^ to (1.02 ± 0.16)­10^6^ M^–1^.^51,52^ Consequently, the change in free energy, which is (−8.46 ± 0.07) kcal/mol for our system, is also comparable.^50–52^

**Figure 7:**
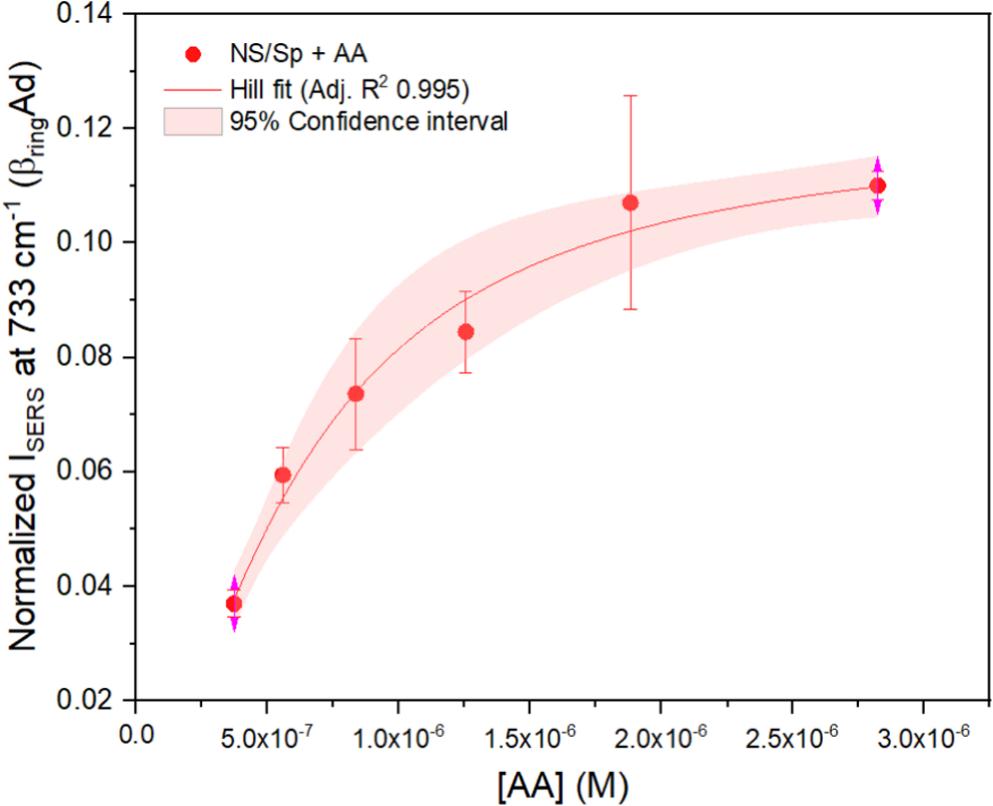
SERS adsorption isotherm of the NS/Sp + AA system. The lowest concentration data of the NS/Sp + AA series shows a Hill-Langmuir behavior (Adj. *R* ^2^ 0.995; Normal residuals), with a *K*_d_ that is 3.5 orders of magnitude higher than that estimated for Sp on NS, and a Δ*G*_ad_ of −8.46 ± 0.07 kcal/mol. Error bars represent the standard error of the means.

**Table 3:** Thermodynamic Parameters for '5-AA-3' Adsorption on Spermine-Capped Nanostars (NS/Sp) at *T* = 20 °C

*K*_d_	(6.2 ± 0.7) 10^–7^ M
*K*_ad_	(1.6 ± 0.2) 10^6^ M^–1^
Δ*G*_ad_	(−8.46 ± 0.07) kcal/mol
cooperativity (*n*_Hill_)	1.5 ± 0.2

The fact that (i) our thermodynamic data for *preadsorbed* spermine and 5′-AA-3′ is of the same order as that reported in the literature for A­(T)-rich longer sequences and the *free* polyamine in solution, and (ii) the SERS spectra of 5′-AA-3′ show an overall analogous profile, both in terms of frequencies and relative intensities, whether they are obtained in the presence of preadsorbed (capping, NS/Sp + AA system) or free (aggregating, NS + AA + Sp system) spermine, suggest that the interaction between spermine and the dinucleotide is the strongest in our system and takes hierarchical precedence over the co-occurring others in the definition of the adsorbate landscape in the context of a colloidal SERS sample. Therefore, an overall relative quantitative relationship among the various pairwise interactions can be drawn:

8 [NS−AA]≈[AA−AA]<[NS−Sp]<[AA−Sp]

where (*i*) the [NS–AA] ≈ [AA–AA] relationship is deduced by the sigmoidal behavior observed for the NS + AA system, (ii) the [AA–AA] < [NS–Sp] relationship is deduced by the observation that spermine disrupts the native sigmoidal behavior of AA in both spermine-containing SERS systems, and (iii) the [NS-Sp] < [AA-Sp] relationship is quantitatively justified by our thermodynamic evaluations.

Future studies based on isothermal titration calorimetry could quantitatively verify all of the hypothesized hierarchies, and further comparative studies leveraging the inclusion of different sequences could aid in the evaluation of additional effects that might come into play when introducing nucleobase-specific modulations of the spermine-DNA interaction. However, since adenine is reported to be the nucleobase with the highest affinity for coinage metals *and* AT-rich sites are reported to have the strongest interactions with spermine,^51,52^ and because the spermine-nucleic acid interaction seems to prevail over the other equilibria, it could be possible that the presence of different nucleobases will not change the trend observed in this work, that sees the spermine-nucleic acid interaction as thermodynamically prevailing.

### 3.5 Effects of Spermine on Analytical Performance

The interaction relationships highlighted in the previous section suggest that DNA sequences analyzed by direct SERS in the presence of spermine interact as spermine-DNA complexes; hence, the effects of spermine on the maximization of the analytical performance should not only be investigated in terms of sensitivity but also *specificity*. While our SERS isotherm data make it apparent that the use of spermine improves the detectability of 5′-AA-3′ and justifies the ubiquity^12–26^ of this polyamine in direct SERS analytical protocols for the detection of nucleic acids (Figure 5, the presence of spermine increases the detectability range by at least ∼1.5 orders of magnitude), the detailed effects of spermine on the ability of the analytical method to yield distinguishable SERS spectra for very minute differences in the sequence of the nucleic acid (e.g., point mutations or methylation) should be as well elucidated. In light of our data, the observations that can be made on the topic of selectivity are very limited, in that the definition of selectivity itself intrinsically requires a comparison between different analytes. However, it can be said that the presence of spermine enriches the SERS spectral features compared to the spectrum obtained in its absence and exhibits frequency shifts at locations that are not confined to the phosphate backbone modes, which are poorly enhanced in SERS, but also encompass spectral regions with significant contributions from the nucleobase’s modes. As a result, it could be hypothesized that the presence of spermine should in principle provide a larger number of nucleobase-sensitive and sequence-sensitive spectral regions, which could increase the selectivity of the method.

On sensitivity grounds, some considerations should also be added. While the order of spermine introduction into the SERS sample had no significant effect on the quality of the SERS spectra, that is, frequencies and *relative* intensities, a drastically different effect was observed on the *overall* signal intensity. The NS/Sp + AA system produced SERS spectra with an overall intensity that was much lower than the one yielded by the NS + AA + Sp system; for example, at a fixed 5′-AA-3′ concentration of (5.9)­10^–6^ M (Figure S14), the ring breathing mode of adenine was 2.6 times more intense when spermine was utilized as an aggregating agent. Consequently, more sensitive limits of detection (LODs) could be expected for sample preparations in which spermine is utilized as an aggregating agent, compared to when it was utilized as a capping agent. While a detailed exploration of sensitivity-related mechanisms is beyond the scope of this paper, a possible explanation for the higher sensitivity associated with the NS + AA + Sp sample preparation might come from aggregation dynamics. A higher degree of aggregation, and thus, interparticle hotspots, might be induced by a reduction of the surface charge of a colloidal entity that is likely more abrupt in the NS + AA + Sp system than in the NS/Sp + AA system, where the modulation of the surface charge of the colloidal entities is operated stepwise with alternatingly charged species (i.e., [anionic NS, cationic Sp, anionic 5′-AA-3′] vs [anionic NS, anionic 5′-AA-3′, cationic Sp]).

## 4 Conclusions

The presented study made use of a unified colloidal nanoparticle system to build a fair comparison platform for the elucidation of the thermodynamic equilibria and relative hierarchy involved in the direct SERS detection of nucleic acids in the presence of spermine. This specific choice of experimental design allowed to eliminate, for the first time, the confounding bias given by the synthesis-dependent nanoparticle surface chemistry that has characterized spermine-focused comparative SERS studies up to now. Moreover, the validation and application of the ssDNA dinucleotide 5′-AA-3′ as a “minimal working example” model analyte allowed to retain all key structural elements of DNA while reducing the large number of degrees of freedom that is usually associated with the longer oligonucleotides. With it, it decreased the electronic size, rendering DFT frequency calculations an accessible aid for in-depth vibrational elucidation.

The use of a thermodynamic framework to conceptualize SERS samples for direct nucleic acid detection allowed for their schematization into three fundamental constituents (i.e., the colloidal metal nanoparticle, the analyte, and spermine) and the identification of a series of pairwise, coexisting adsorption equilibria. Taking advantage of the fair comparison platform given by the colloidal gold silver nanostars that are utilized for the study, the pairwise equilibria were experimentally decoupled and characterized. The resulting combined thermodynamic and spectroscopic data suggest that the interaction with spermine disrupts the native adsorption mechanism of 5′-AA-3′ individually adsorbed on the nanostars, via the introduction of a new interaction in the system, which is the strongest among the co-occurring ones, as demonstrated by the fact that the SERS frequencies and their relative intensities appear essentially independent from the order of addition of the sample components. A summarizing relative ranking of the energetics characterizing the ternary SERS system were therefore drawn to follow [NS – AA] ≈ [AA – AA] < [NS – Sp] < [AA – Sp], with adsorbed spermine and free spermine having analogous behavior toward the nucleic acid. These conclusions are in contrast with the main previous comparative study on the effect of different SERS sample preparation for the direct detection of nucleic acids in the presence of spermine.^19^ This previous study, however, does not benefit of our unified nanoparticle platform approach, and we ascribe such divergences to a possible direct consequence of experimental design differences, and thus, to the presence or elimination of the confounding bias associated with nanoparticle surface chemistry. Implementing experimental designs that are conscious of the confounding bias imposed by synthesis-driven nanoparticle surface chemistry are important beyond SERS, as many nanotechnological applications, ranging for example from drug formulation and delivery to other types of sensing, are sustained or modulated by surface adsorption phenomena.^76,108,138^

From a SERS-specific point of view, the fundamental information that has been collected and presented herein is intended to be utilized as one of the grounds on which to develop powerful methods that can keep pushing the boundaries of bioanalytical SERS. On this matter, we are witnessing a growing number of multivariate analysis- and machine learning (ML)-powered bioanalytical SERS applications,^139–144^ because the inherent complexity of biological systems produces highly complex SERS spectra whose information is undeniably more powerfully leveraged by ML than with traditional interpretation pipelines. However, substantial criticism is often raised toward the “black box” character of many artificial intelligence methods for data treatment, for which the decisional process remains opaque, hindering both true and perceived reliability.^145^

To establish SERS for rigorous and reliable routine bioanalysis, nonblack-box methods should be developed,^26,146^ and this approach should equally include both input *and* output sides of the generation of scientific information. If on one hand nonblack-box machine learning algorithms should be pushed forward and their application routinized,^26,146^ on the other hand, their performance and output will only be as powerful as the input data enable them to be. Therefore, elucidating the *physical meaning* of SERS spectra of biological analytes through fundamental studies like the one here presented will also intrinsically and synergistically contribute to promoting the development of nonblack-box ML-assisted SERS implementations, whereby futuristic avenues such as recognizing the identity *and* location of genetic biomarker mutations via direct SERS analysis^26^ can become an actualizable breakthrough.

## Supplementary Material



## Notes

Additional Raman and SERS spectra of the investigated systems, including background spectra, individual spectra of AA at different excitation wavelengths, spectra of mixtures modeled as linear combinations of the individual components, and comparisons between different analyte concentrations; a representative extinction spectrum of the nanostars, with and without spermine; TEM and STEM micrographs of the nanostars with EDX mapping; plots of residuals of the Hill-Langmuir fit of ELS data; a pictorial representation of the possible origin of a sigmoidal-looking SERS adsorption isotherm; additional tables reporting the spectral resolution of our Raman microscopy system, as well as the temperature, viscosity, relative permittivity, and refractive index utilized for the ELS study; Cartesian coordinates of 5′-AA-3′ (charge = 0, multiplicity = 1) optimized at the B3LYP/6-311G** level of theory; supporting equations S1 and S2, describing the Debye–Hueckel parameter and the Bjerrum length (PDF)
