# Supplementary material for: Direct SERS Detection of Nucleic Acids in the Presence of Spermine: A Unified Nanoparticle Platform Allows for the Elucidation of Surface Adsorption Hierarchies
Source: J Phys Chem C Nanomater Interfaces. 2025 May 21;129(22):10163–80. doi: 10.1021/acs.jpcc.5c01965 (PMC12147209; doi:10.1021/acs.jpcc.5c01965)
Supplement: Supplementary file 1 [file jp5c01965_si_001.pdf]

Direct SERS Detection of Nucleic Acids in the Presence of Spermine:
A Unified Nanoparticle Platform Allows for the Elucidation of Surface Adsorption Hierarchies

Chiara Deriu[1] and Laura Fabris*[1]

[1] Department of Applied Science and Technology, Politecnico di Torino, 10129 Turin, Italy

SUPPORTING INFORMATION

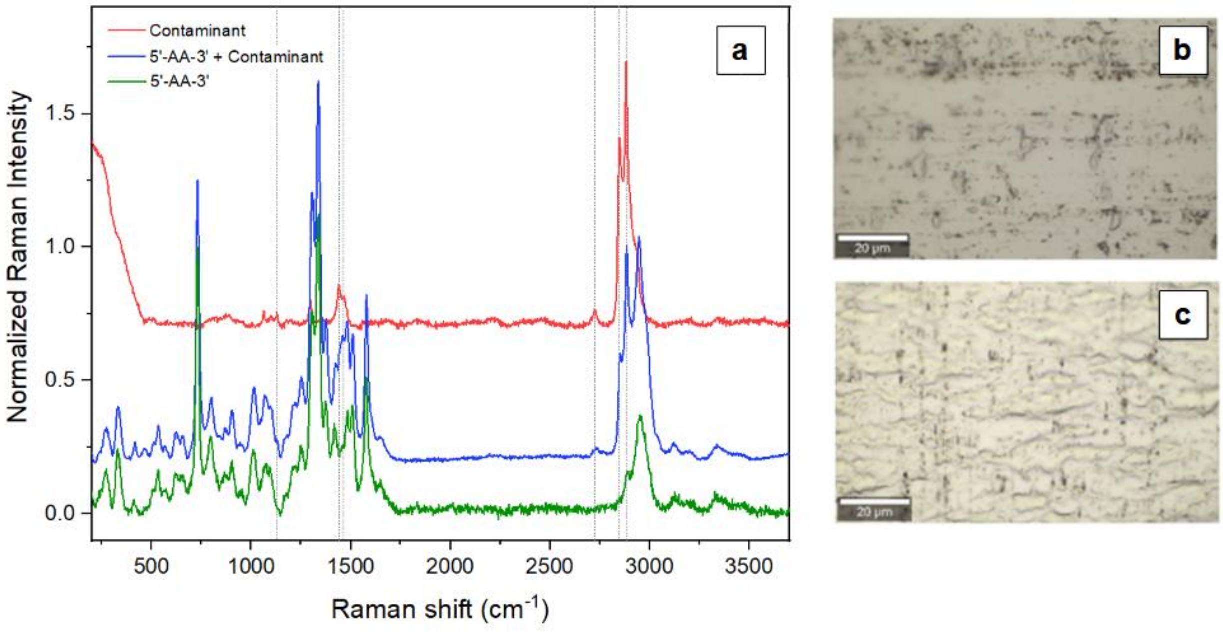

Figure S1. Presence of a lipidic contaminant on aluminum foil. *(a)* Raman spectra of the dried residue of a dinucleotide, 5'-AA-3', obtained after prior drop casting of the analyte's aqueous solution on an untreated (blue spectrum) and on a plasma-cleaned (green spectrum) piece of aluminum foil. The blue spectrum in the middle is different from the bottom, green spectrum, due to the presence of a contaminant (top, red spectrum). The contaminant was identified to be of lipidic nature, likely a mixture of triglycerides, phosphatidic acids, and sphyngomyelins.[1] *(b)* Optical microscope image of an untreated piece of aluminum foil. Clear and dark residues are visible. *(c)* Optical microscope image of a plasma-cleaned piece of aluminum foil. The wettability of the surface increased, the apparent roughness increased, the clear residues disappeared, and some dark residues remained. Small traces of amorphous carbon are associated with the dark residues, which are found not to interfere with the spectral profile of the investigated analytes. Spectra are stacked for ease of observation.

Table S1. Spectral resolution across the 200–2000 cm^{-1} range for the WITec Apyron system fitted with a 300 g/mm grating.

λ_{exc} (nm)	Reference wavenumber (cm^{-1})						spectral resolution across the 200–2000 cm^{-1} range
	200	500	700	1000	1500	2000	
**532**	3.01	2.91	2.85	2.75	2.60	2.44	3.01–2.44 cm^{-1}
**633**	2.11	2.03	1.98	1.90	1.77	1.67	2.11–1.67 cm^{-1}
**785**	1.88	1.79	1.73	1.64	1.50	1.36	1.88–1.36 cm^{-1}

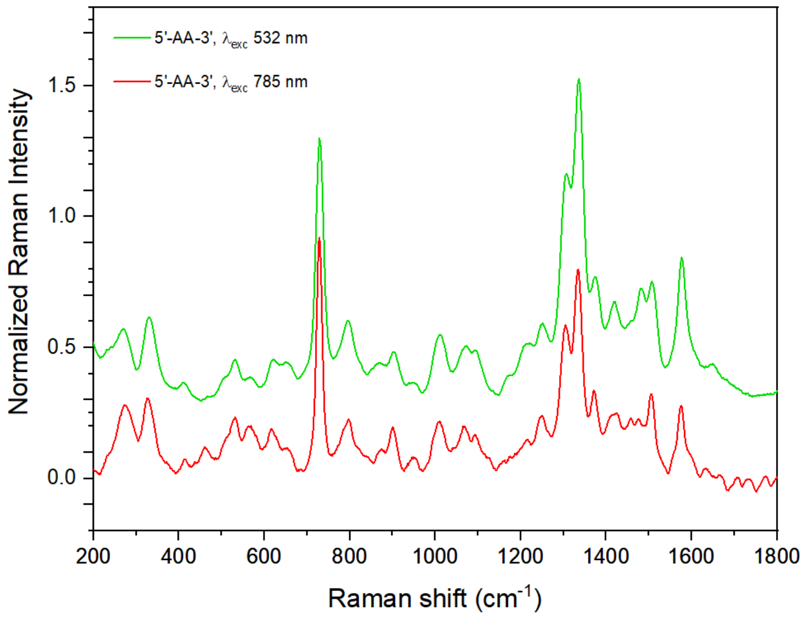

Figure S2. Raman spectra of the model ssDNA dinucleotide 5'-AA-3' solid state residues, acquired with two different excitation lines: 532 nm (*top, green*) and 785 nm (*bottom, red*). Apart from slight signal to noise ratio (S/N) differences, no dissimilarities are observed in terms of band position or relative intensities with varying excitation line. While this spectral profile homogeneity was expected due to a lack of resonance conditions between the analytes' electronic transitions at the utilized excitation wavelengths (*i.e.*, oligonucleotides electronic transitions fall in the range between 190 and 260 nm), it can also be added that, at the explored illumination conditions (excitation wavelength, optical power, and the total exposure time), 5'-AA-3' does not seem to be subjected to photoinduced modifications. Possible spectral profile deviations from the obtained traditional Raman references that might be observed in our SERS experiments are therefore to be restricted to the presence of the nanoparticle surface and/or to interaction with spermine. Spectra are stacked for ease of observation.

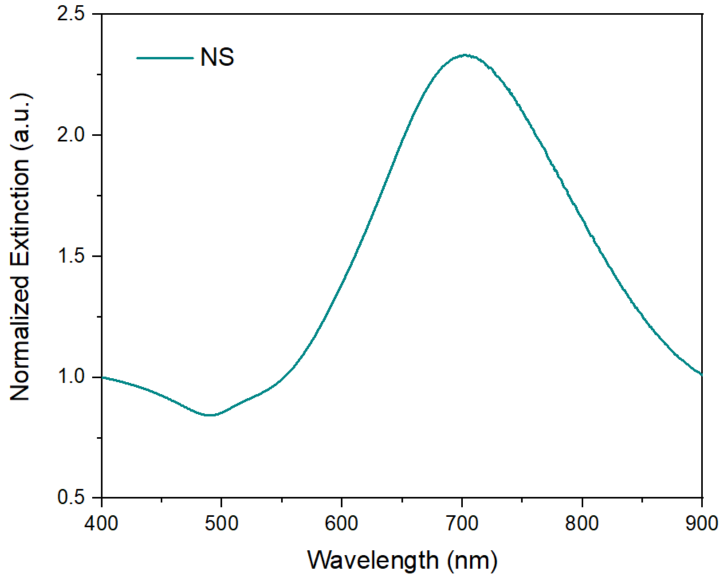

Figure S3. Representative extinction spectrum of the employed nanostars. The localized surface plasmon resonance (LSPR) maximum is centered at around 700 nm.

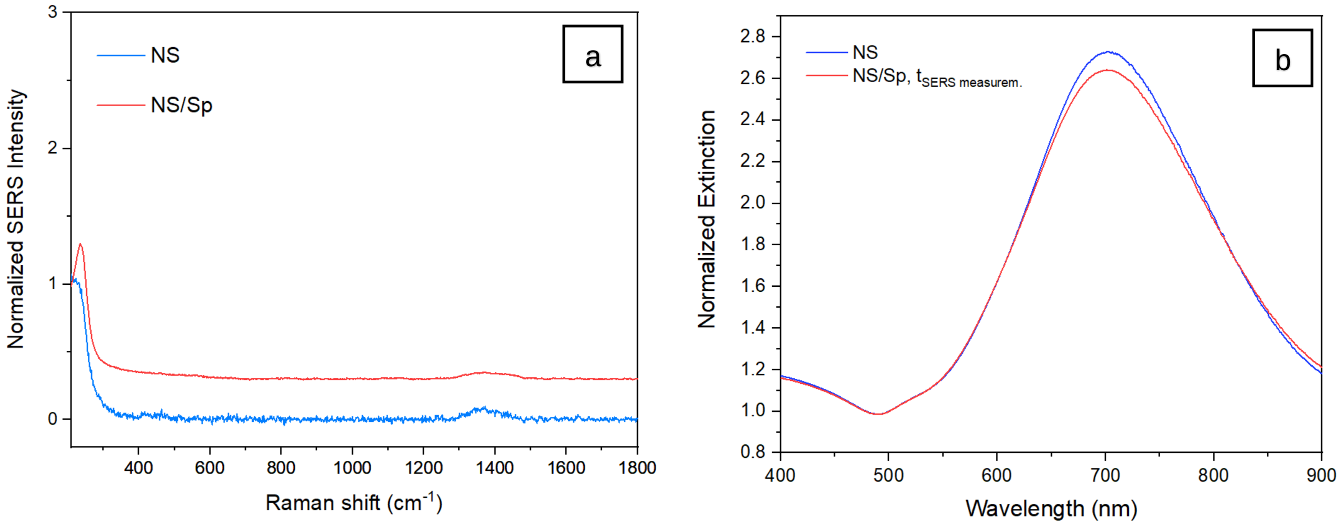

Figure S4. *(a)* Representative SERS background spectra of the as-synthesized nanostars (NS, blue spectrum) and of the same nanostars capped with spermine (NS/Sp, red spectrum). The spectra have been baseline-corrected and normalized by the intensity of the metal–Cl stretching band at ~230 cm^{-1}. *(b)* Representative extinction spectra of the as-synthesized nanostars (NS, blue spectrum) and of the same nanostars capped with spermine (NS/Sp, red spectrum), acquired within the timeframe of a SERS measurement (about 3 minutes). The added dielectric medium provided by sperimine is minimal, and as such, it does not cause significant shifts to the LSPR maximum. The spectra have been normalized by their intensity at 400 nm.

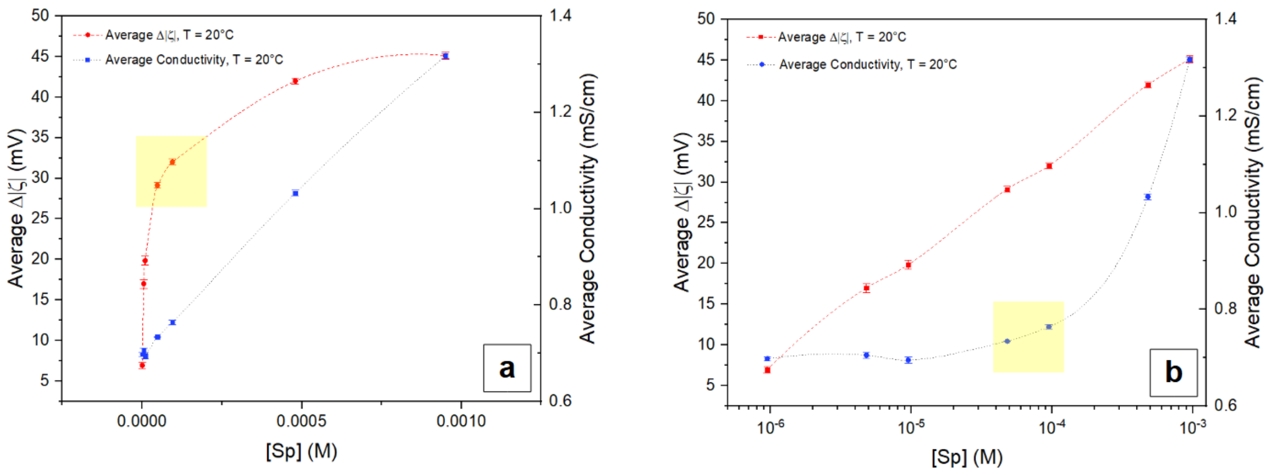

Figure S5. Electrophoretic light scattering (ELS) titration data (T = 20 °C) of spermine tetrahydrochloride (Sp) on colloidal nanostars (red circles) and the corresponding bulk conductivity of the colloid at each titration point (blue squares) shown on *(a)* a linear and *(b)* a logarithmic concentration scale. A concentration of $(6.7)10^{-5}$ M was chosen as the optimized spermine concentration because it falls at the inflection point (highlighted in yellow) of the titration curve. Following the validated methodology developed in Deriu *et al.*,[2] at the inflection point of an ELS titration curve of a nanoparticle system that exhibits a Langmuir-like behavior, the capping agent is approaching monolayer saturation. At such concentrations of capping agent, the nanoparticle is endowed with sufficient electrostatic repulsion to have colloidal stability. At the same time, the concentration of the capping agent is not at saturation, thus avoiding easy destabilization with added adsorbing species, such as an analyte to be measured by SERS. Consequently, these capping agent concentration conditions prevent fast and uncontrollable aggregation during SERS measurement. Concomitant measurements of the bulk conductivity of the colloid at each titration point shows that the same concentrations around the inflection point of the Δ|ζ| curve correspond to the inflection point of the conductivity curve itself (highlighted in yellow and better visualized on a $\log_{10}$ scale in *panel b*). In Langmuir-like charged adsorbate-nanoparticle systems, conductivity grows exponentially when monolayer saturation is approached: The charged titrant starts to populate the bulk of the solution instead of adsorbing on the surface of the nanoparticle, thus increasing the system's conductivity. When the conductivity of the bulk of the solution grows, so does its ionic strength, which progressively compresses the electric double layer of the colloidal nanoparticle, thus increasing colloidal instability. Error bars represent the standard error of the mean. Akima splines are utilized to guide the reader's eye.

Table S2. Temperature, Viscosity,[3] relative permittivity,[4] and refractive index (λ = 667 nm)[5] utilized for the ELS SOP.

T (°C)	T (K)	*η* H_2O	*ε* H_2O	*n* H_2O
20	293.15	1.0016	80.10	1.331

η, viscosity; *ε*, dielectric permittivity; *n*, refractive index.

Equations S1 and S2. Debye-Hueckel parameter [m^{-1}] and Bjerrum length [m].[6]

$$\kappa = 4\pi\lambda_B \sum(n_{c_i} z_i) \quad \text{(S1)}$$

Where λ_B is the Bjerrum length, n_{Ci} is the number concentration of the ith charged species, and z_i is the corresponding formal charge.

$$\lambda_B = \frac{q_e^2}{4\pi\varepsilon_r\varepsilon_0 k_B T} \quad \text{(S2)}$$

Where q_e is the elementary charge, ε_r and ε_0 are the relative permittivity of the dielectric medium and the permittivity of vacuum, respectively, and k_B is the Boltzmann constant. At T = 20 °C, λ_B is 7.12 x 10^{-10} m.

Table S3. Cartesian Coordinates of 5'-AA-3' (charge = 0, multiplicity = 1) optimized at the B3LYP/6-311G** level of theory.

C	-2.815433	2.227308	-1.156212
C	-3.912339	2.638645	-0.159126
C	-5.185603	2.101181	-0.816334
C	-4.722187	0.856506	-1.573053
O	-3.333679	1.081889	-1.876804
O	-4.004500	4.041143	0.005093
C	-1.507474	1.787626	-0.530013
O	-1.022526	2.881736	0.285414
P	0.549856	2.930193	0.610566
O	1.459377	3.403500	-0.449328
O	0.533191	3.839823	1.936052
O	0.802112	1.436541	1.137165
C	2.004760	0.691198	0.820341
C	2.970274	0.677232	2.008598
C	3.567395	-0.738539	2.005945
O	2.687577	-1.551711	1.248799
C	1.638652	-0.792299	0.622490
C	1.560245	-1.277382	-0.822813
O	0.353100	-0.778668	-1.447816
P	-0.798915	-1.794963	-1.928674
O	-0.353672	-3.065581	-2.518437
O	-1.656159	-1.924978	-0.556940

O	-1.680324	-0.897596	-2.903187
N	6.899493	0.325313	-1.840732
C	5.614086	0.659702	-1.676605
N	4.803268	0.367349	-0.655881
C	5.427394	-0.350004	0.284826
C	6.756370	-0.773040	0.253149
C	7.502220	-0.397019	-0.882126
N	4.926064	-0.829657	1.481911
C	5.971447	-1.521949	2.071859
N	7.078639	-1.506113	1.380915
N	8.803067	-0.727582	-1.032080
N	-5.004949	-1.835966	2.922127
C	-5.060089	-0.517908	2.700784
N	-5.024186	0.134696	1.536111
C	-4.916913	-0.699839	0.497689
C	-4.841888	-2.090230	0.574643
C	-4.891119	-2.655519	1.864983
N	-4.872490	-0.413241	-0.863115
C	-4.753256	-1.639091	-1.496070
N	-4.725787	-2.660862	-0.681177
N	-4.814103	-3.986936	2.072213
H	-2.653525	3.047273	-1.862396
H	-3.762276	2.139293	0.802674
H	-5.971087	1.893468	-0.093801
H	-5.539267	2.842727	-1.536470
H	-5.237032	0.728249	-2.525756
H	-3.225212	4.321427	0.497971
H	-1.665133	0.916704	0.108485
H	-0.775596	1.534110	-1.298905
H	1.089154	4.618782	1.817625
H	2.471813	1.114738	-0.066303
H	3.732222	1.452731	1.946339
H	2.391680	0.823023	2.923206
H	3.629334	-1.147937	3.017151
H	0.690026	-0.997398	1.129811
H	2.405469	-0.900565	-1.398402
H	1.574334	-2.367816	-0.841668
H	-2.351188	-2.598422	-0.613060
H	-2.210450	-0.177619	-2.501761
H	5.175229	1.243057	-2.479981

H	5.838425	-2.014121	3.024795
H	9.240120	-1.348259	-0.372319
H	9.258068	-0.510979	-1.902953
H	-5.147422	0.101467	3.588086
H	-4.670681	-1.702031	-2.571635
H	-4.826405	-4.621059	1.291502
H	-4.923070	-4.336546	3.009512

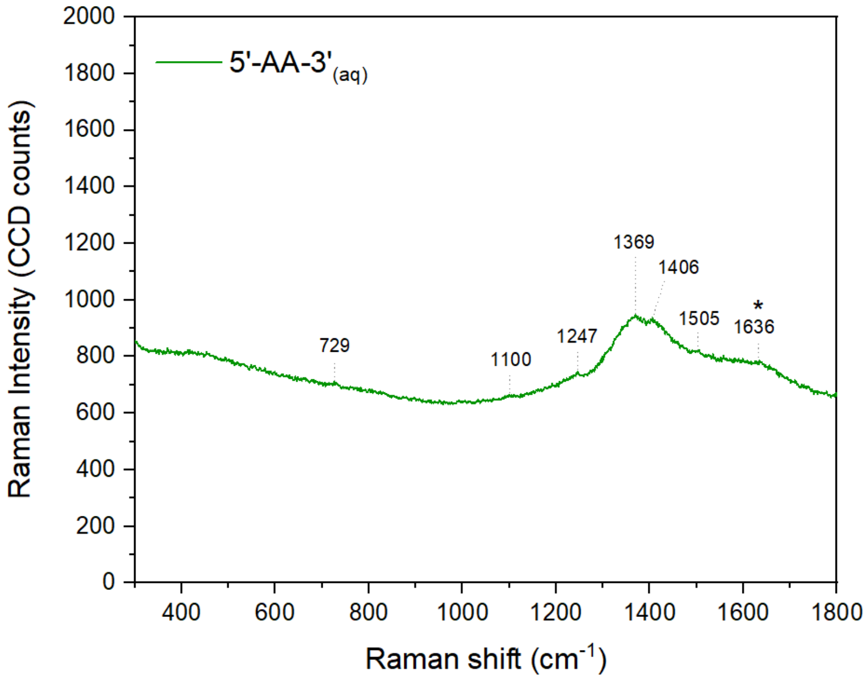

Figure S6. Raman spectrum of 5'-AA-3' in solution, at a concentration of (1.14)10^{-3} M. The signal was obtained using the optimized parameters: λ_{exc} 785 nm, 54 mW, 5 s, 10 accumulations. The H–O–H bending mode of water is marked with an asterisk.

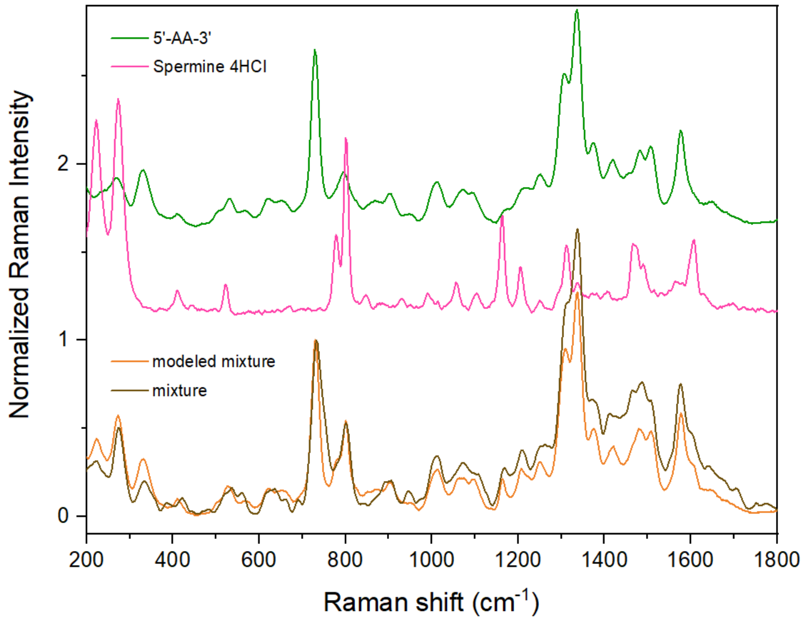

Figure S7. Comparison among the Raman spectra of 5’-AA-3’ and spermine tetrachlorohydrate in the solid state (*top*, green and pink, respectively) with their mixture, also in the solid state (*bottom*, brown spectrum). The mixture cannot be modeled by linearly combining the spectra of the two individual components (*bottom*, orange spectrum). Spectra are varyingly stacked for ease of observation.

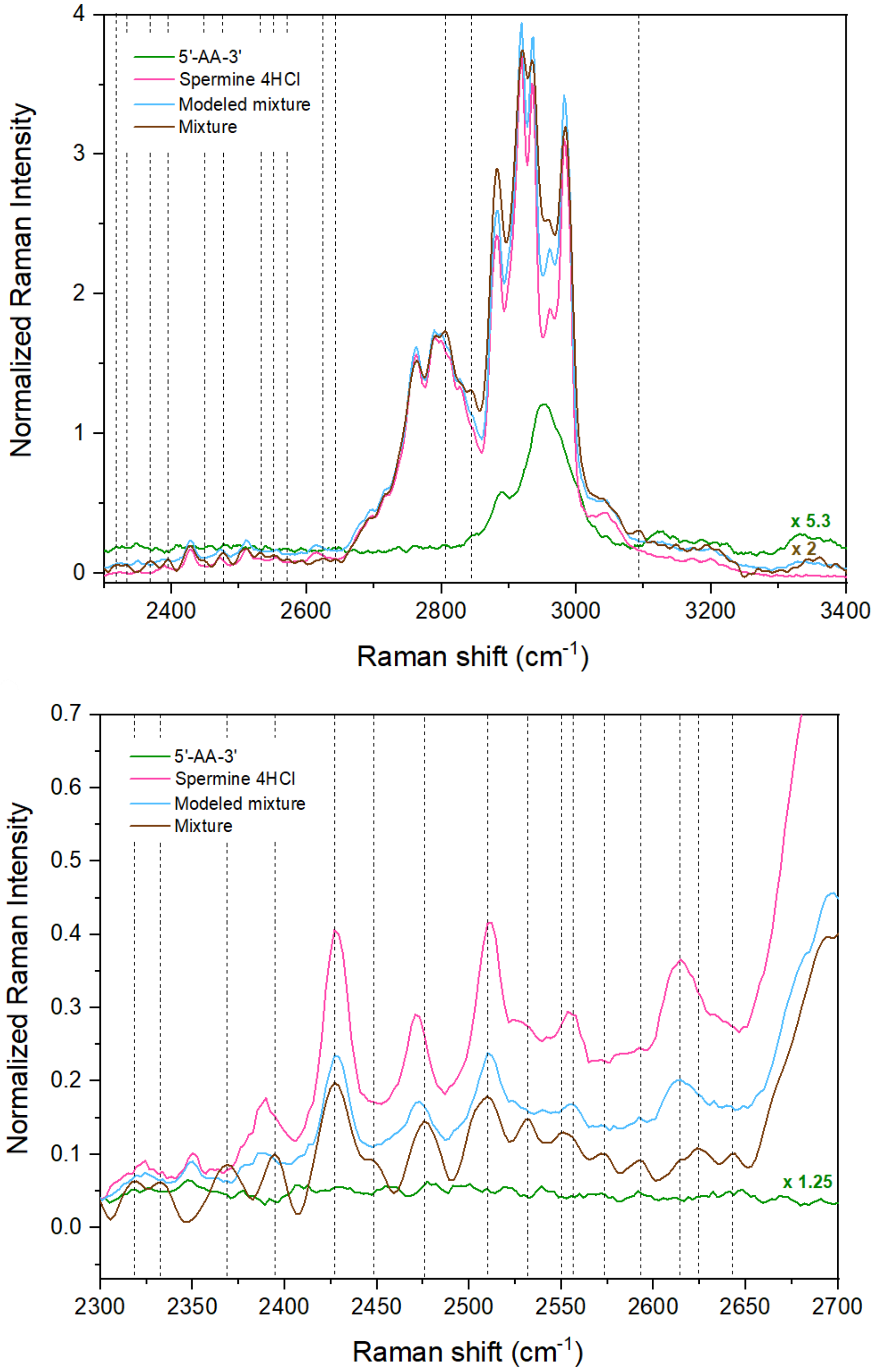

Figure S8. Comparison among the Raman spectra of 5'-AA-3' and spermine tetrachlorohydrate in the solid state (green and pink spectra, respectively) with their mixture (brown spectrum), also in the solid state. Even in this higher wavenumber region, the mixture cannot be modeled by linearly combining the spectra of the two individual components (light blue spectrum). Dashed vertical lines are added to guide the eye and locate the bands of the mixture. Where reported, Raman intensities have been multiplied by a factor to allow for a clearer visualization of the traces.

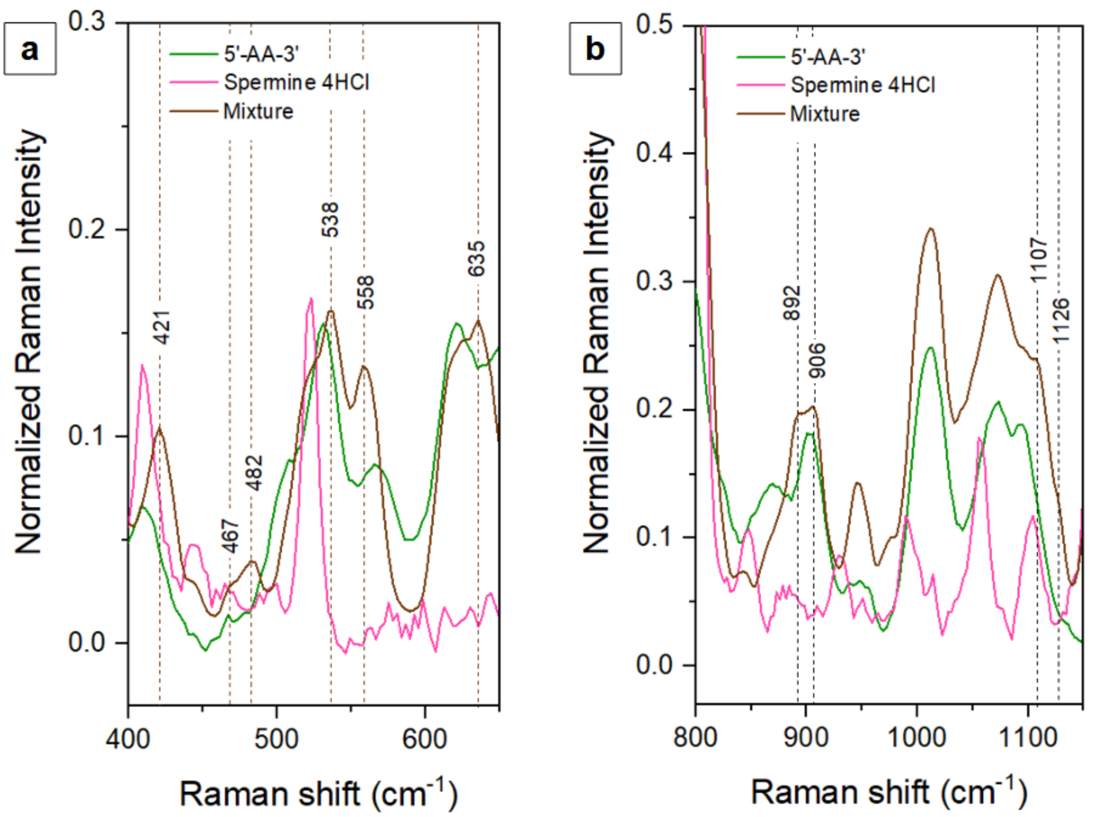

Figure S9. Raman spectra (λ_{exc} 532 nm) of the dried residues of 5’-AA-3’ (*green*), spermine tetrahydrochloride (*pink*), and their mixture (*brown*). (a) Zoomed in regions between 400 and 650 cm^{-1} and (b) between 800 and 1150 cm^{-1}. All intensities have been normalized to the most intense band in the 700–850 cm^{-1} region (~730 cm^{-1} for both 5’-AA-3’ and the mixture, and 800 cm^{-1} for spermine tetrahydrochloride; see *Figure 3* in the main manuscript for the whole range spectrum) and are expressed in arbitrary units.

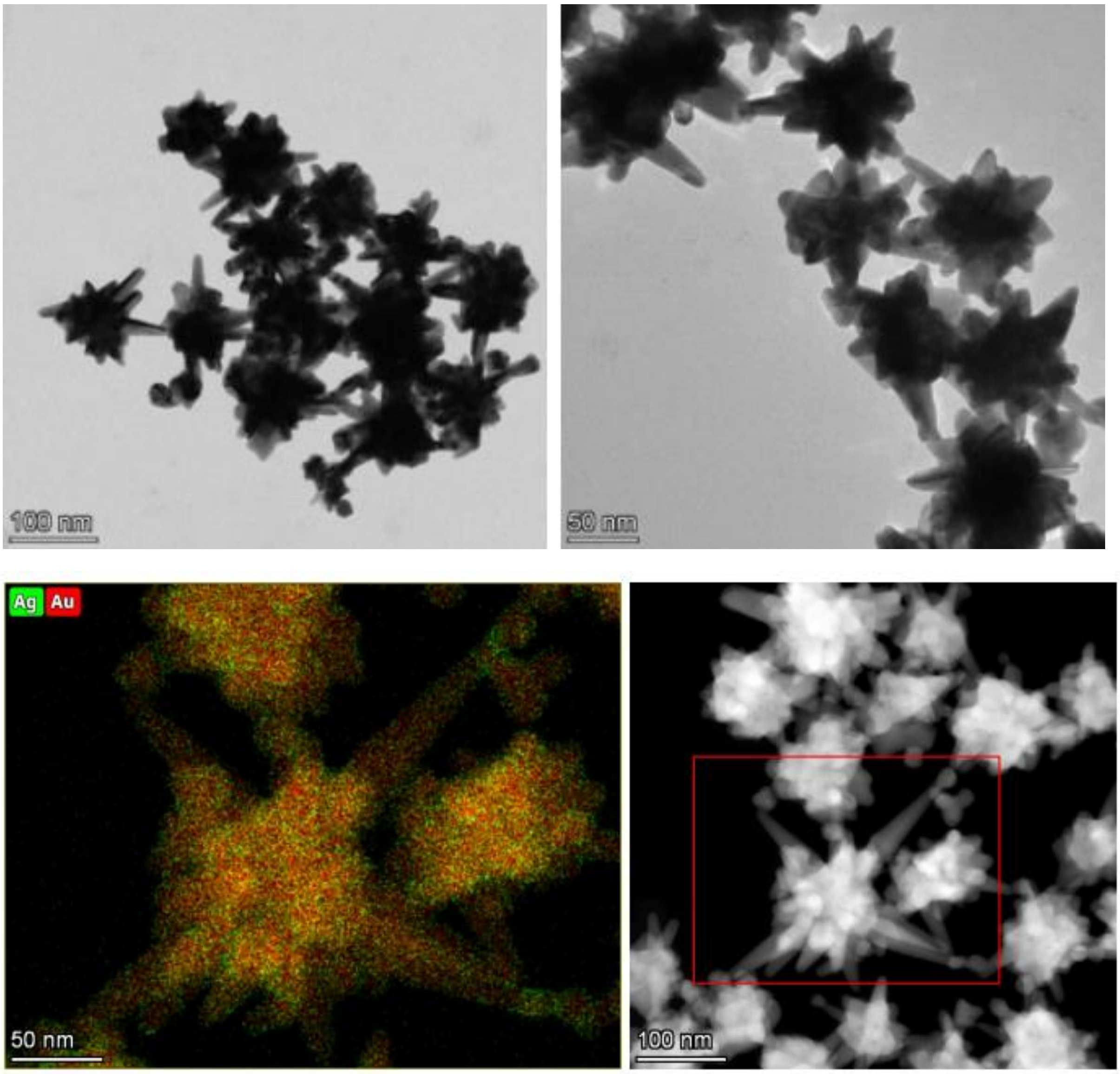

Figure S10. Electron microscopy characterization of the employed nanostars, before centrifugation. (*top*) Bright field TEM micrographs, (*bottom*) EDX mapping (*right*), and STEM micrograph (*left*). The EDX map shows silver in green and gold in red, with no clear signs of compositional segregation.

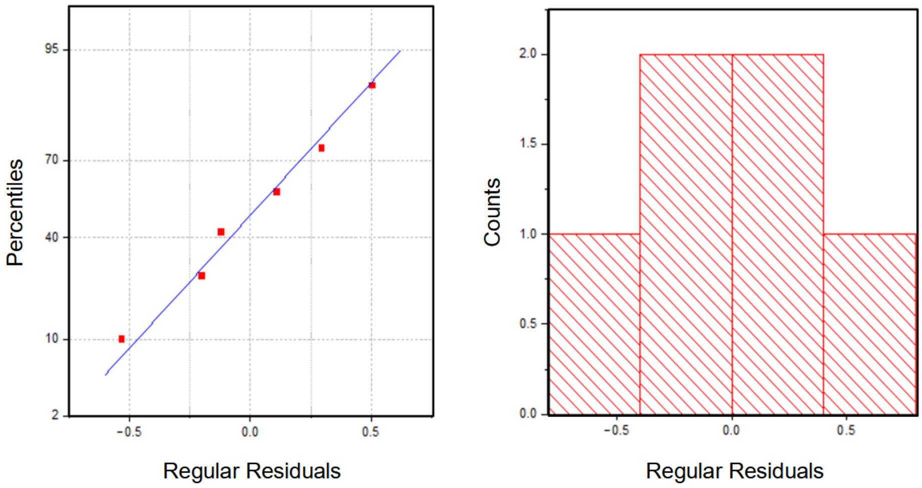

Figure S11. Plots of residuals obtained from the Hill-Langmuir fit of the ELS-derived adsorption isotherm of spermine on the colloidal nanostars. The residuals are normally distributed, thus supporting the goodness of fit of the Langmuir-Hill model for these ELS data.

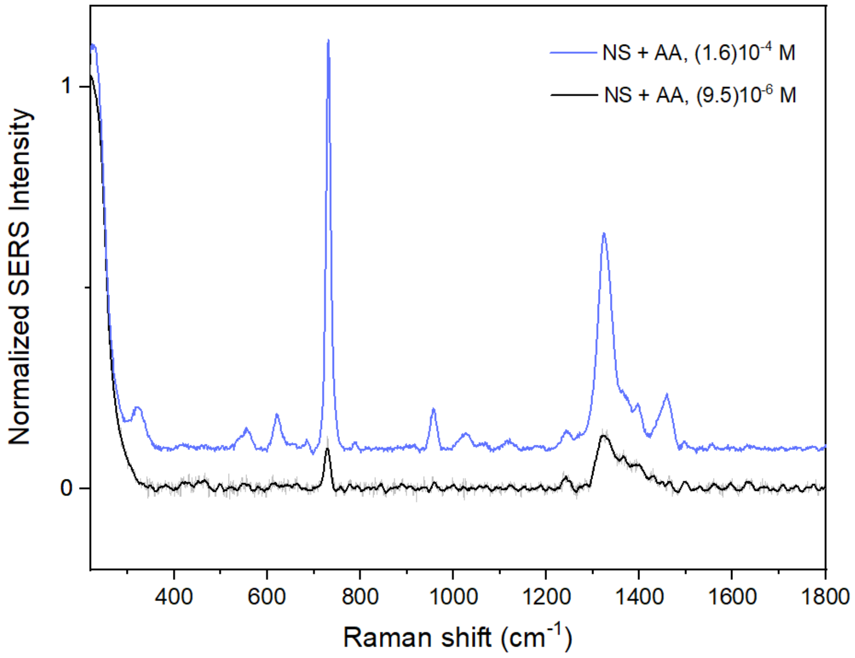

Figure S12. Comparison between the NS + AA SERS spectra at a higher concentration (*top*, blue) and at a lower concentration (*bottom*, black). To maximize clarity, the lower concentration spectrum was smoothed with a Savitzky-Golay filter (15 pts window and 2nd order polynomial). The original, noisier trace is shown in gray for transparency. The bands that keep emerging in the lower concentration spectrum are consistent with a uniform, concentration-dependent decrease of the overall SERS intensity. No other intensity-related effects are noticed. Spectra are stacked for ease of observation.

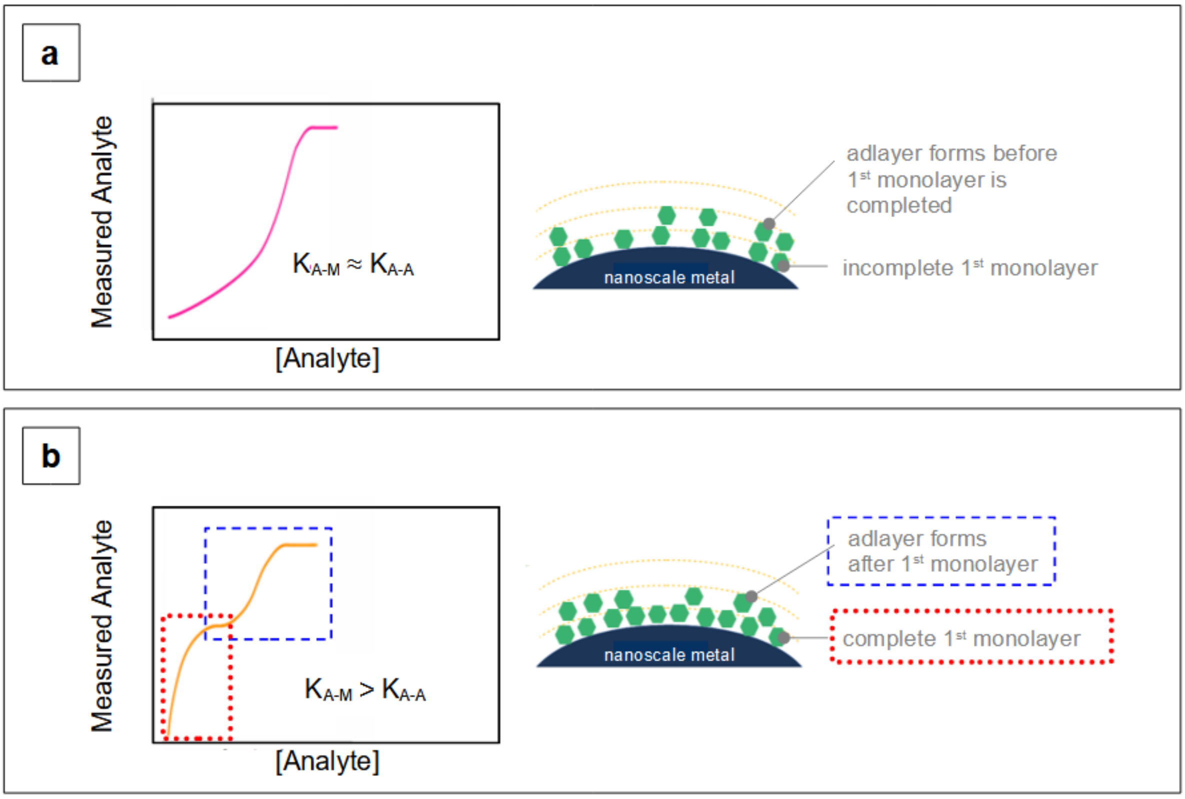

Figure S13. Pictorial representation of the possible origin of a sigmoidal-looking adsorption isotherm. (*a*) A sigmoidal isotherm typically results from the formation of adlayers before a complete monolayer is established, due to the similarity between the analyte-metal affinity constant and the analyte-analyte affinity constant ($K_{A\text{-}M} \approx K_{A\text{-}A}$). The sigmoidal behavior resulting from such a thermodynamic scenario is a "true sigmoidal" behavior, otherwise known as a class S isotherm, according to Giles[7,8] classification system. (*b*) Apparent sigmoidal behaviors could also result from the formation of adlayers after saturation of the first monolayer (L4 isotherm according to Giles[7,8] classification system). This type of isotherm could be mistaken for a true sigmoidal isotherm *if* data are acquired within the blue dashed area (higher analyte concentration range only). In this case, the initial, Langmuir-like behavior is lost (red, dotted area in the graph), compromising the accurate interpretation of the results.

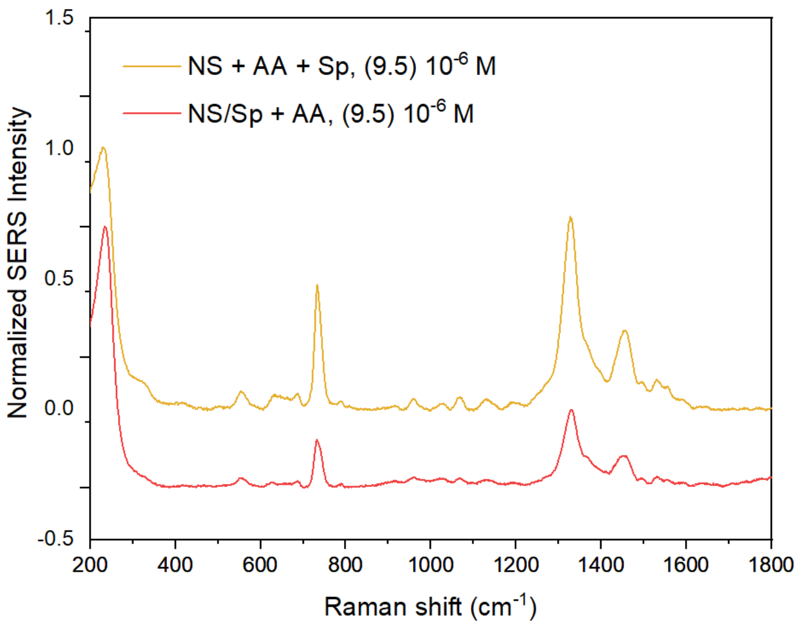

Figure S14. Comparison between the NS + AA + Sp (*top*, orange) and NS/Sp + AA SERS spectra (*bottom*, red), obtained at the same concentration of (9.5)10^{-6} M. While the relative intensity relationships and frequencies are equivalent, the overall SERS intensity is noticeably higher for the NS + AA + Sp system. For example, the intensity of the ring breathing mode of adenine at ~ 730 cm^{-1} is 2.6 times higher. Spectra are stacked for ease of observation.

REFERENCES

(1) Krafft, C.; Neudert, L.; Simat, T.; Salzer, R. Near Infrared Raman Spectra of Human Brain Lipids. *Spectrochim Acta A Mol Biomol Spectrosc* **2005**, *61* (7), 1529–1535. https://doi.org/10.1016/j.saa.2004.11.017.

(2) Deriu, C.; Bracho, A.; McCord, B. Tailored Colloidal Nanostars for Surface Enhanced Raman Spectroscopy (SERS): Optimization of Formulation Components and Study of the Stabilizer-Nanoparticle Interactions. *The Journal of Physical Chemistry C* **2022**, *126* (4), 2023–2040. https://doi.org/10.1021/acs.jpcc.1c08145.

(3) The International Association for the Properties of Water and Steam (IAPWS). *Release on the IAPWS Formulation 2008 for the Viscosity of Ordinary Water Substance*; Berlin, Germany, 2008.

(4) Malmberg, C. G.; Maryott, A. A. Dielectric Constant of Water from 0°to 100°C. *J. Res. Natl. Bur. Stand.* **1956**, *56* (1), 2641–1–2641–2645.

(5) Bashkatov, A. N.; Genina, E. A. Water Refractive Index in Dependence on Temperature and Wavelength: A Simple Approximation. In *Proceedings of SPIE Vol. 5068, Laser Physics and Photonics, Spectroscopy, and Molecular Modeling III; Coherent Optics of Ordered and Random Media III, Fall 2002*; Tuchin, V. V., Ed.; Society of Photo-Optical Instrumentation Engineers (SPIE): Saratov, Russian Federation, 2003; pp 393–395.

(6) LeRu, E. C.; Etchegoin, P. *Principles of Surface-Enhanced Raman Spectroscopy*; Elsevier, 2009. https://doi.org/https://doi.org/10.1016/B978-0-444-52779-0.X0001-3.

(7) Giles, C. H.; MacEwan, T. H.; Nakhwa, S. N.; Smith, D. Studies in Adsorption. Part XI. A System of Classification of Solution Adsorption Isotherms, and Its Use in Diagnosis of Adsorption Mechanisms and in Measurement of Specific Surface Areas of Solids. *J Chem Soc* **1960**, No. 0, 3973–3993. https://doi.org/10.1039/jr9600003973.

(8) Giles, C. H.; Smith, D.; Huitson, A. A General Treatment and Classification of the Solute Adsorption Isotherm. I. Theoretical. *J Colloid Interface Sci* **1974**, *47* (3), 755–765. https://doi.org/10.1016/0021-9797(74)90252-5.
